# Tissue Tropism in Host Transcriptional Response to Members of the Bovine Respiratory Disease Complex

**DOI:** 10.1038/s41598-017-18205-0

**Published:** 2017-12-20

**Authors:** Susanta K. Behura, Polyana C. Tizioto, JaeWoo Kim, Natalia V. Grupioni, Christopher M. Seabury, Robert D. Schnabel, Laurel J. Gershwin, Alison L. Van Eenennaam, Rachel Toaff-Rosenstein, Holly L. Neibergs, Luciana C. A. Regitano, Jeremy F. Taylor

**Affiliations:** 1 Division of Animal Sciences, University of Missouri, Columbia, MO United States of America; 2 Embrapa Southeast Livestock, São Carlos, SP Brazil; 3 Departamento de Ciencias Exatas, UNESP - Univ Estadual Paulista, Faculdade de Ciencias Agrarias e Veterinarias, Jaboticabal, SP 14884-900 Brazil; 4 Department of Veterinary Pathobiology, College of Veterinary Medicine, Texas A&M University, College Station, TX United States of America; 5 Informatics Institute, University of Missouri, Columbia, MO United States of America; 6 Department of Pathology, Microbiology & Immunology, School of Veterinary Medicine, University of California, Davis, California, United States of America; 7 Department of Animal Science, College of Agriculture, University of California, Davis, CA United States of America; 8 Department of Animal Sciences, College of Agriculture and Natural Resource Sciences, Washington State University, Pullman, WA United States of America

## Abstract

Bovine respiratory disease (BRD) is the most common infectious disease of beef and dairy cattle and is characterized by a complex infectious etiology that includes a variety of viral and bacterial pathogens. We examined the global changes in mRNA abundance in healthy lung and lung lesions and in the lymphoid tissues bronchial lymph node, retropharyngeal lymph node, nasopharyngeal lymph node and pharyngeal tonsil collected at the peak of clinical disease from beef cattle experimentally challenged with either bovine respiratory syncytial virus, infectious bovine rhinotracheitis, bovine viral diarrhea virus, *Mannheimia haemolytica* or *Mycoplasma bovis*. We identified signatures of tissue-specific transcriptional responses indicative of tropism in the coordination of host’s immune tissue responses to infection by viral or bacterial infections. Furthermore, our study shows that this tissue tropism in host transcriptional response to BRD pathogens results in the activation of different networks of response genes. The differential crosstalk among genes expressed in lymphoid tissues was predicted to be orchestrated by specific immune genes that act as ‘key players’ within expression networks. The results of this study serve as a basis for the development of innovative therapeutic strategies and for the selection of cattle with enhanced resistance to BRD.

## Introduction

Bovine respiratory disease (BRD) is the most common infectious disease of beef and dairy cattle and is responsible for 70–80% of morbidities and 40–50% of feedlot cattle mortalities in the United States^1^. BRD is a multifactorial disorder with a complex infectious etiology involving several viral and bacterial pathogens collectively referred to as the bovine respiratory disease complex (BRDC). Disease is frequently initiated by environmental stresses such as transportation to a feedlot, which can predispose susceptible calves to a primary infection, usually by a viral pathogen, which depresses the host immune system facilitating a secondary infection of the lower respiratory tract by bacteria^2,3^. Clinical diagnosis can vary according to the specific combination of causal pathogens but is usually based on the manifestation of lethargy or depression, reduced feed intake, fever, increased respiratory rate and dyspnea. Consequently, diagnosis is often made without the identification of disease etiology, leading to an incomplete diagnosis of undifferentiated bovine respiratory disease^4^. Treatment with broad-spectrum antibiotics requires the early recognition of disease to assist in the recovery of animals, although animal productivity is often extremely compromised in those that recover^4^. Many protection methods have now emerged, however, vaccines protect only about 75% of vaccinated animals and do not provide defense in epidemics where the causal pathogens differ from those targeted by the vaccines^4^. Consequently, vaccines and antimicrobials have not been effective in decreasing BRD morbidity or mortality rates and BRD remains prevalent despite being widely studied^5^.

The exact pathogenic mechanisms by which bacteria and viruses cause BRD and the suite of factors that contribute to development of disease are not fully understood^6^. Pathogen infection triggers a dynamic cascade of immune response events that result in perturbed gene expression patterns^7^. RNA sequencing (RNA-Seq) allows the investigation of global gene expression changes and is repeatable, highly sensitive and strongly correlated with quantitative polymerase chain reaction experiments^8^. We have previously employed RNA-Seq to examine the bronchial lymph node transcriptomes of beef steers exposed to single agents of the BRDC^9^, however, the examination of diverse tissue transcriptomes can provide greater insights into the role of variation in transcript abundance as it relates to infectious disease susceptibility. Though, in general, infections by bacterial or viral pathogens in animals infect multiple types of cells and tissues, some may primarily infect a single tissue and facets of the immune response may be tissue specific^10^. Thus, the interaction of pathogen with host tissue niches is an important factor determining tissue tropism that reflects the ability of a given pathogen to infect a specific tissue and the level of host defense that a tissue can elicit towards the invading pathogen. In this study, we examined the mRNA expression changes in multiple lymphoid tissues as well as lung in beef cattle experimentally challenged individually with different bacterial or viral pathogens of the BRDC. The primary aim of this investigation was to examine how tissue cooperative transcriptional responses are modulated upon challenge by BRDC pathogens to produce a normal immune response to infection. Also of considerable interest was the identification of the genes that are the key drivers of the normal transcriptional response to infection, since mutations within these genes or the regulatory regions that control the expression of these genes have the potential to impair the immune response.

## Results

### Tissue gene expression profiles

**Figure 1:**
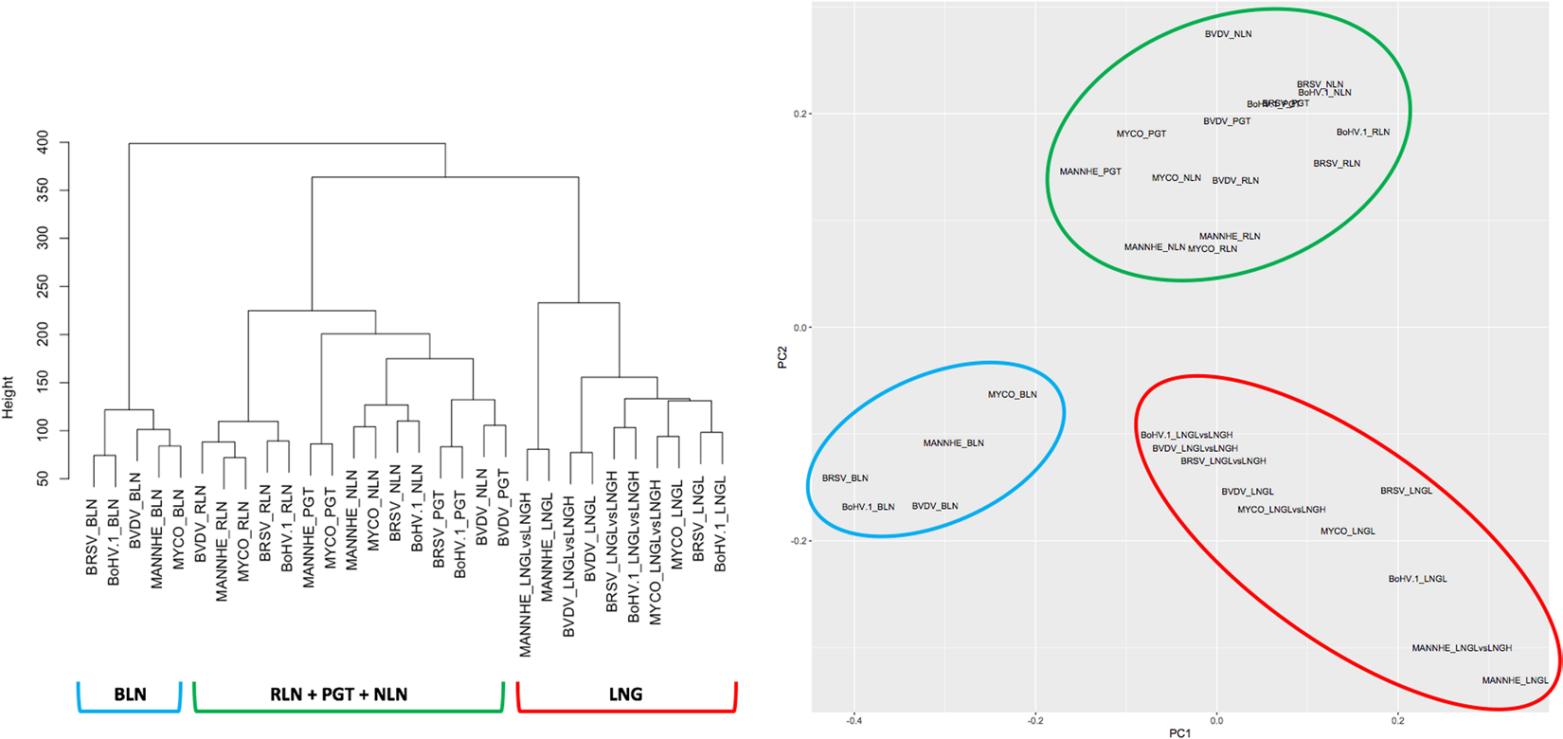
(**A**) Hierarchical cluster tree, and (**B**) Principal component analysis of gene expression changes across tissues and challenge pathogens. The cluster groups for each pathogen and tissue combination are indicated by colored lines below the branch nodes in the cladogram (lung, LNG includes contrasts between challenge and control groups and lesion and healthy lung tissue from the same animal) and are circled in the principal component plot.

The RNA-Seq experiments generated, on average, ~50 million reads per sample. The overall read alignment rate ranged from 84.8% to 94.1% and concordant pair mapping rate ranged from 74.2% to 93.1% (Supplementary Table 1). Transcript abundance differences between challenged and control animals were estimated for samples derived from lung lesion (LNGL) and four lymphoid tissues: bronchial lymph node (BLN), retropharyngeal lymph node (RLN), nasopharyngeal lymph node (NLN) and pharyngeal tonsil (PGT). For the lung, we also determined transcript abundance differences between LNGL and a nearby sample devoid of lesions collected from the lung (LNGH) of the same animal, with all tissue samples being collected at the peak of clinical signs. The gene expression changes in each tissue due to experimental challenge by individual pathogens (Bovine Respiratory Syncytial Virus, BRSV; Bovine Viral Diarrhea Virus, BVDV; Bovine herpesvirus 1, BoHV-1; *Mannheimia haemolytica*, MANNHE; or *Mycoplasma bovis*, MYCO) revealed variation in transcriptional profiles that were clustered into only 3 groups by both hierarchical clustering and principal component analysis (Fig. 1). The first two principal components captured 22.7% and 13.9% of the variance, respectively, in gene expression across all of the samples. The expression changes in BLN in response to challenge by the different BRDC pathogens clustered into a group that was distinct from other groups. Similarly, the gene expression changes identified in lung lesion samples relative to either unchallenged control animal lung samples or apparently healthy lung samples from the same animal (and therefore exposed to the same challenge pathogen) clustered into a second distinct group. Finally, gene expression changes in the RLN, NLN and PGT tissues clustered into the third group (Fig. 1).

### Differentially expressed genes in response to BRDC pathogens

The RNA-Seq data analysis revealed differential gene expression in different tissues between control and challenged animals, as well as between healthy lung and lung lesion samples from the same animals. The expression values along with significance (p-value and q-value) for the tests of differential expression are provided in the Supplementary Dataset. Different numbers of differentially expressed (DE) genes were identified for each of the tissue × pathogen combinations with fewer genes DE in the animals challenged with the bacterial pathogens than those challenged with viruses (*p* < 0.00001; Table 1). Across pathogens, the RLN had, on average, the fewest and BLN had the greatest number of DE genes. However, variation in the number of DE genes across pathogens was higher for RLN and NLN compared to the other tissues suggesting a greater specificity of pathogen response for these tissues. The number of DE genes differed across the tissue × pathogen combinations (*p* < 0.00001) with far more than the expected numbers of genes DE in BLN and LNGL in response to *M. haemolytica*, NLN and PGT in response to BVDV, and RLN in response to BRSV (Table 1). Table 2 shows the number of genes found to be DE in all 31 combinations of tissues ranging from a single tissue to all 5 analyzed tissues. Among the multi-tissue response genes, there were many more than the expected number of genes DE in both PGT and RLN in response to BRSV, in BLN, NLN and RLN in response to BoHV-1, in NLN and PGT in response to BVDV, in BLN and LNGL in response to *M. haemolytica*, and LNGL and NLN in response to *M. bovis*. The proportion of genes that were DE in two or more tissues was similar for all three viral pathogens (0.49–0.52) and both bacterial challenges (0.20–0.25). However, the proportion for bacterial challenges combined is less than half that of the combined viral challenges (2 × 2 contingency table; *p* < 0.00001), suggesting that gene expression in response to bacterial infections is largely tissue-specific.

**Table 1:** Numbers of differentially expressed genes for each tissue and pathogen combination*.

	BLN	LNGL	NLN	PGT	RLN	Mean	Std Dev
BRSV	3484	3129	1577	2472	4337	2999.8	1041.9
BVDV	3317	1580	4381	3945	1269	2898.4	1401.9
BoHV-1	4122	3117	3340	1838	2799	3043.2	832.1
MANNHE	2352	2195	196	530	616	1177.8	1014.0
MYCO	328	906	994	734	157	623.8	365.5
Mean	2720.6	2185.4	2097.6	1903.8	1835.6	2148.6	—
Std Dev	1480.1	969.8	1722.1	1391.9	1718.4	—	1390.3

*The differentially expressed genes were determined by comparing transcript abundance differences between challenged and control (unchallenged animals) for each pathogen and each tissue. Abbreviations used: bronchial lymph node (BLN), retropharyngeal lymph node (RLN), nasopharyngeal lymph node (NLN), pharyngeal tonsil (PGT) and lung lesion (LNGL). The pathogens: bovine respiratory syncytial virus (BRSV), Bovine herpesvirus 1 (BoHV-1), bovine viral diarrhea virus (BVDV), *Mannheimia haemolytica* (MANNHE) and *Mycoplasma bovis* (MYCO).

**Table 2:** Numbers of differentially expressed genes in common between tissues or specific to a single tissue.

Tissues	BRSV	BoHV-1	BVDV	MANNHE	MYCO
BLN	924	1371	1244	1395	131
LNGL	1247	1247	606	1342	610
NLN	156	819	901	97	675
PGT	394	334	936	327	480
RLN	1187	279	66	249	59
BLN-LNGL	294	343	102	564	57
BLN-NLN	46	150	333	14	29
BLN-PGT	36	68	139	55	13
BLN-RLN	670	453	117	148	15
LNGL-NLN	65	244	101	19	89
LNGL-PGT	117	117	94	56	53
LNGL-RLN	248	62	8	59	11
NLN-PGT	102	184	1268	7	93
NLN-RLN	43	170	66	9	7
PGT-RLN	448	19	14	10	5
BLN-LNGL-NLN	27	93	37	9	15
BLN-LNGL-PGT	17	20	30	31	7
BLN-LNGL-RLN	312	181	29	80	9
BLN-NLN-PGT	27	52	406	3	11
BLN-NLN-RLN	86	417	189	10	7
BLN-PGT-RLN	189	63	31	14	8
LNGL-NLN-PGT	65	82	262	3	29
LNGL-NLN-RLN	14	78	28	4	3
LNGL-PGT-RLN	107	13	3	3	0
NLN-PGT-RLN	135	87	108	1	6
BLN-LNGL-NLN-PGT	19	14	84	1	5
BLN-LNGL-NLN-RLN	82	192	40	10	3
BLN-LNGL-PGT-RLN	106	27	12	10	2
BLN-NLN-PGT-RLN	301	354	414	5	9
LNGL-NLN-PGT-RLN	61	80	34	1	6
BLN-LNGL-NLN-PGT-RLN	348	324	110	3	7
Total DE Genes Across Tissues	7873	7937	7812	4539	2454

Footnote for abbreviations: bronchial lymph node (BLN), retropharyngeal lymph node (RLN), nasopharyngeal lymph node (NLN), pharyngeal tonsil (PGT) and lung lesion (LNGL). For LNGL the comparison is to lung samples from uninfected control individuals. The pathogens: bovine respiratory syncytial virus (BRSV), Bovine herpesvirus 1 (BoHV-1), bovine viral diarrhea virus (BVDV), *Mannheimia haemolytica* (MANNHE) and *Mycoplasma bovis* (MYCO).

**Figure 2:**
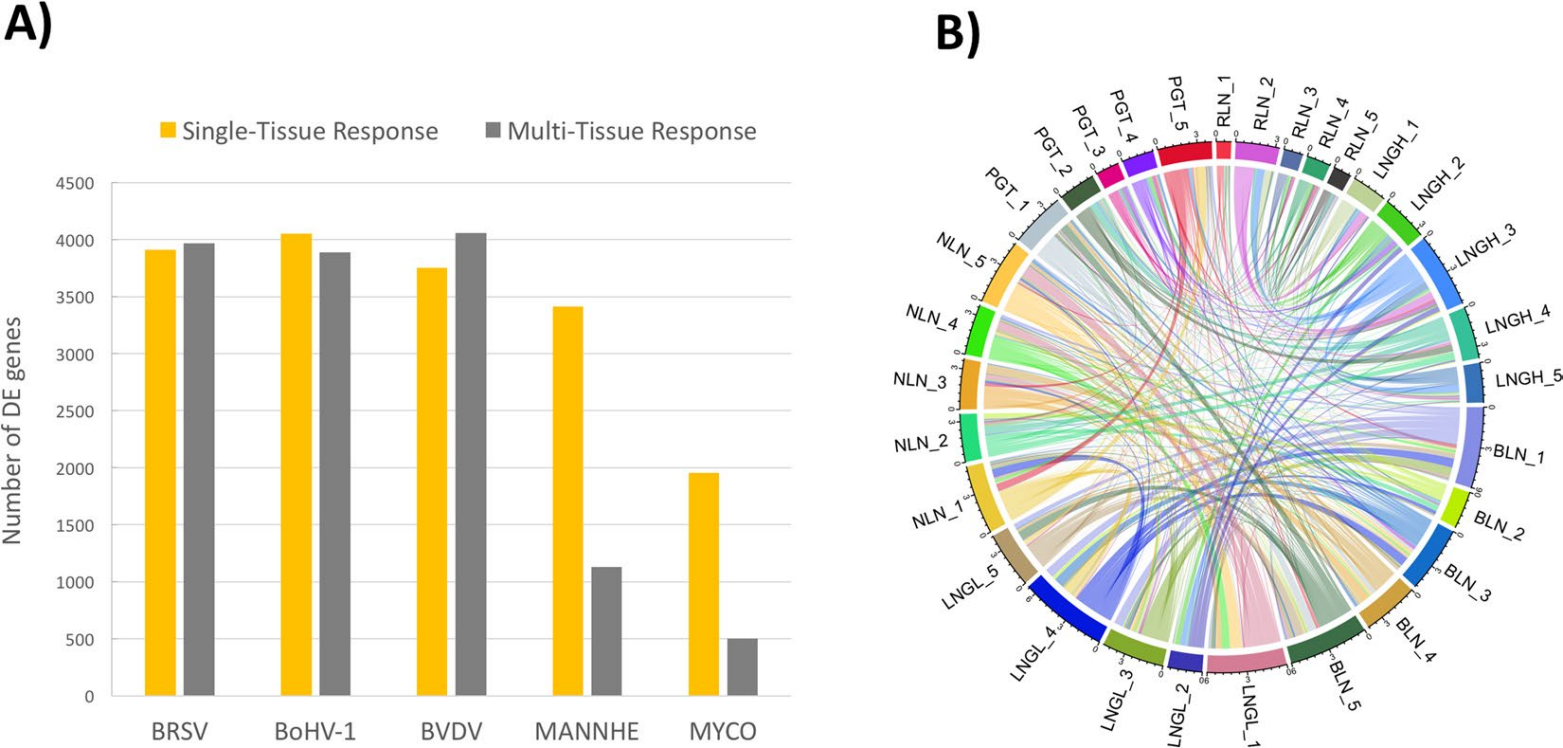
Numbers of differentially expressed genes in tissues. (**A**) Number of differentially expressed genes that respond to each pathogen challenge in a single tissue or in multiple tissues. (**B**) A chord diagram showing the inter-relationships of significant changes in gene expression among different tissues (BLN, LNGL, LNGH, NLN, PGT and RLN) to challenge by the different pathogens (1 = BRSV, 2 = BVDV, 3 = BoHV-1, 4 = MANNHE and 5 = MYCO).

**Figure 3:**
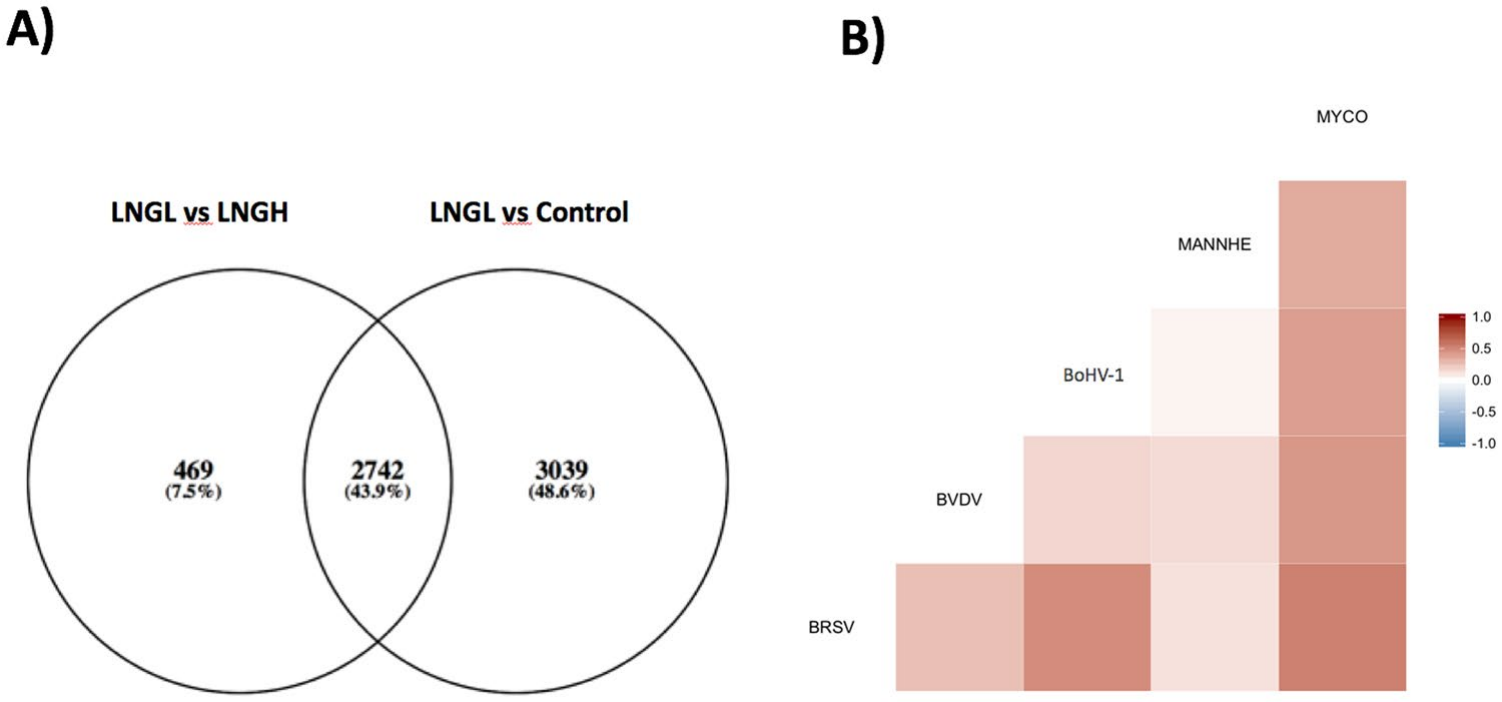
(**A**) Venn diagram indicates that about 44% of differentially expressed genes are in common across all pathogens in the LNGL *versus* Control and LNGL *versus* LNGH comparisons. (**B**) Pair-wise correlations between gene expression changes between LNGL *versus* LNGH relative to challenge pathogens.

**Figure 4:**
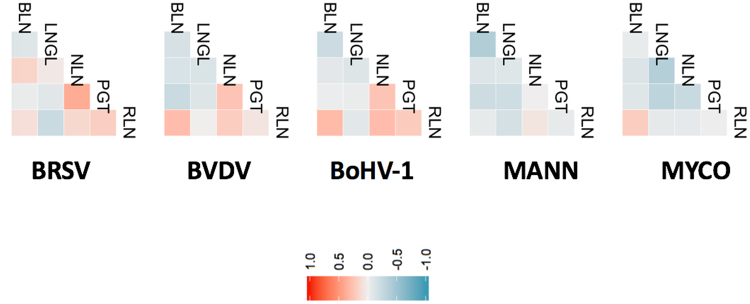
Pair-wise correlations between gene expression changes between pairs of tissues in response to challenge by the different BRDC pathogens. Color codes are relative to the indicated correlation scale.

The complexity of the relationships between DE genes among tissues is shown in Fig. 2B in a chord diagram. This map provides a graphical representation of the mutual information (a non-linear measure of dependency) of expression changes among genes across all tissue × pathogen combinations. Thus, Fig. 2B represents the extent of relationships between host transcriptional responses in different tissues that are due to the different pathogens. Among the DE genes identified in LNGL relative to LNGH, ~85% (2742 of 3211) were also found to be DE in the comparison of LNGL to the uninfected lungs of the control animals (Fig. 3A). In comparing lung lesion to healthy lung, we observed that the *M. haemolytica* challenge elicited a host transcriptome response that was the least related to the host transcriptional responses to the other pathogens (Fig. 3B). This may be due to the relatively larger changes in gene expression that occurred in response to *M. haemolytica* than for the other pathogens. Furthermore, the correlations between gene expression changes between pairs of tissues within each pathogen challenge group again revealed the tissue specificity of immune response to the bacterial challenges relative to the viral challenges (Fig. 4). In particular, the sign of the correlation between gene expression responses between PGT and NLN appears to discriminate between bacterial and viral infections. This is consistent with the fact that the immune response in these two tissues failed to distinctly cluster across the challenge pathogens (Fig. 1).

The Ingenuity Pathway Analysis software indicated that the majority of the DE genes were involved in pathways related to antimicrobial response, mostly innate, but also adaptive. In the challenged animals we generally found the up-regulation of pathways for acute phase signaling, complement system, regulation of cytokine production, interleukin and interferon signaling, granulocyte and agranulocyte adhesion and diapedesis, as well as the predominant down-regulation of a few lipid and cholesterol metabolism-related pathways such as peroxisome proliferator-activated receptor (PPAR) signaling, liver X receptor (LXR)/retinoid X receptor (RXR) activation and farnesoid X receptor (FXR)/RXR activation and antioxidant action of vitamin C. Likely downstream effects on cellular and organismal biology, such as the stimulation of lymphocyte activating factor IL1B, were also predicted from the expression data. We regularly predicted immunological, inflammatory and respiratory diseases as well as inflammatory responses. These findings suggest that we captured transcriptional variation within these tissues that were indicative of the organismal changes induced by infection.

Genes responsible for nonspecific defense mechanisms against all respiratory disease pathogens^11^ such as those encoding mucins, pattern recognition receptors (PPRs), host defense peptides (such as defensins, lactotransferrin and secretory leukoprotease inhibitor), and matrix metallopeptidase family members were consistently found to be DE across all challenge groups and tissues as were genes with reactive oxygen and wound healing (coagulation factors, *THBD* and *VWF*) functions. Among the pathways most enriched for DE genes across all pathogens, we consistently observed the activation of acute phase response signaling. While for most pathogens Oncostatin M (*OSM*) and *IL1* appeared to regulate the expression of acute phase proteins, TNFα was induced by the *M*. *haemolytica* challenge. We also observed an enrichment of DE genes in pathways related to IL8 signaling, leukotriene, zymosterol, estrogen and cholesterol biosynthesis, eicosanoid signaling, hypercytokinemia and inhibition of matrix metalloproteases but the status of stimulation (up- or down-) could not always be predicted.

We hypothesized that in addition to a general immune response, pathogen-specific immune responses might be elucidated by sequencing the global RNA profiles of tissues collected at the peak of clinical disease^12^. We found many DE genes that were exclusive to each challenge group that were related to specific immune responses. While genes that were exclusively found to be DE between BRSV challenged and control animals, regardless of tissue, were involved in oxidative phosphorylation, mitochondrial dysfunction and hypoxia, the genes found to be exclusively DE in response to BoHV-1 infection were primarily involved in pathways involved in geranylgeranyl diphosphate biosynthesis via mevalonate, glutamate receptor signaling, mevalonate pathway, serotonin receptor and TGFβ signaling. The genes uniquely DE in response to BVDV challenge, were primarily related to the Ephrin receptors, which may function as entry receptors for BVDV. Pathways related to inhibition of viral replication such as Eukaryotic Initiation Factor 2 (EIF2) and Cell Cycle: G1/S Checkpoint Regulation, were also found to be enriched for the genes that were uniquely DE in the BRSV challenged animals. Genes related to T-cell differentiation and/or activation were induced by BVDV challenge. While immune responses to the viral challenges appear to be similar, involving Th1, Th2 and Th17 cells and the subsequent production of interferon, interleukins and immunoglobulins, immune responses to *M. haemolytica* and *M. bovis* appear to be primarily driven by Th2, with the subsequent production of IL1 in addition to TNFα for *M. haemolytica*. Whereas the interferons and their receptors appear to be major regulators of viral immune response, lipopolysaccharide, IL1β and TNF are the major regulators of responses activated by infection with *M. haemolytica* or *M. bovis*.

### Signatures of tissue-specific gene expression

**Figure 5:**
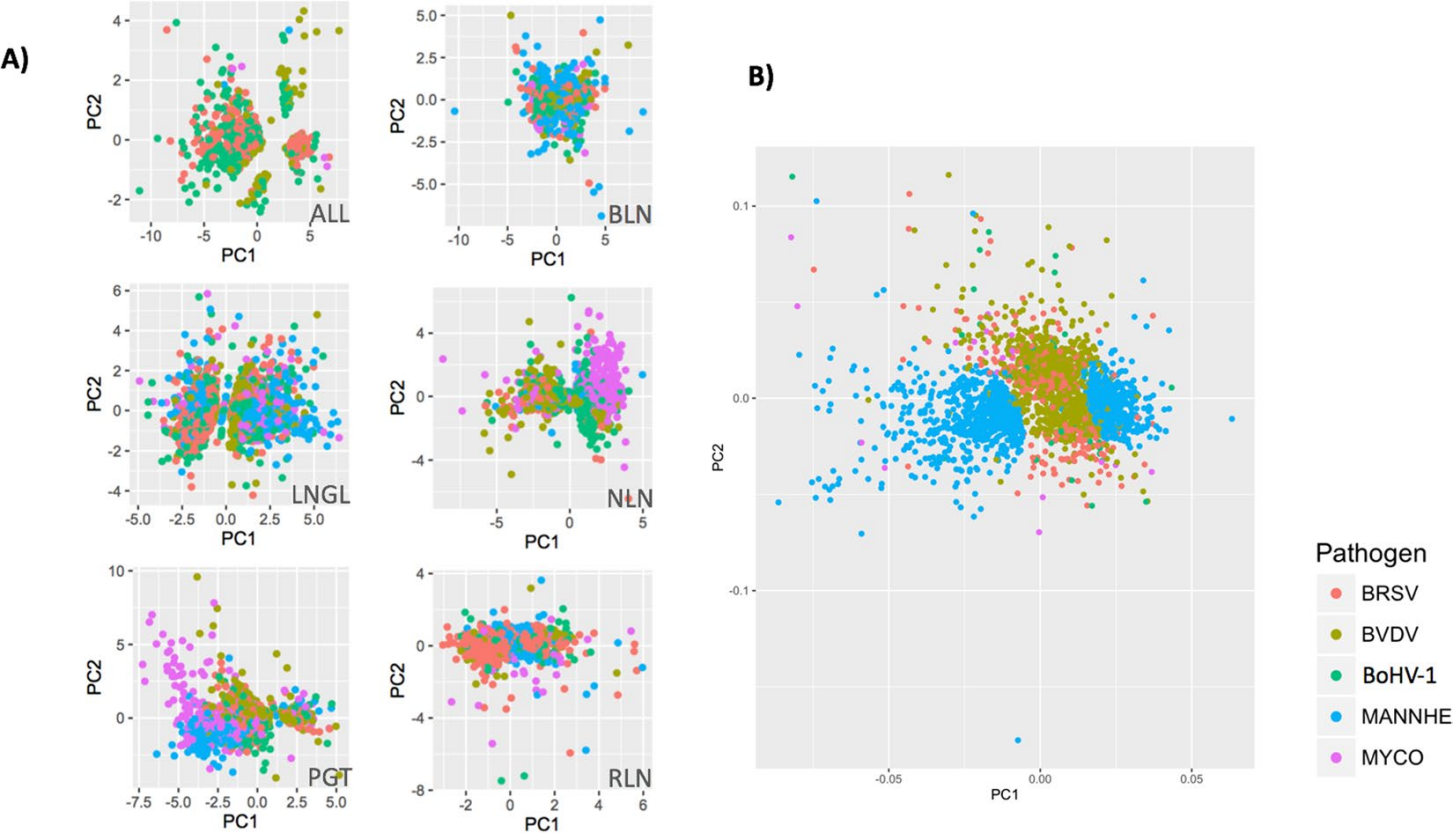
(**A**) Principal component analysis of gene expression for genes differentially expressed in either a single tissue (identified by tissue code) or ubiquitously in all tissues (identified as ‘ALL’) for each challenge pathogen. (**B**) Principal component analysis of genes differentially expressed between LNGL and LNGH in response to individual pathogens. Color codes corresponding to pathogens are shown.

**Figure 6:**
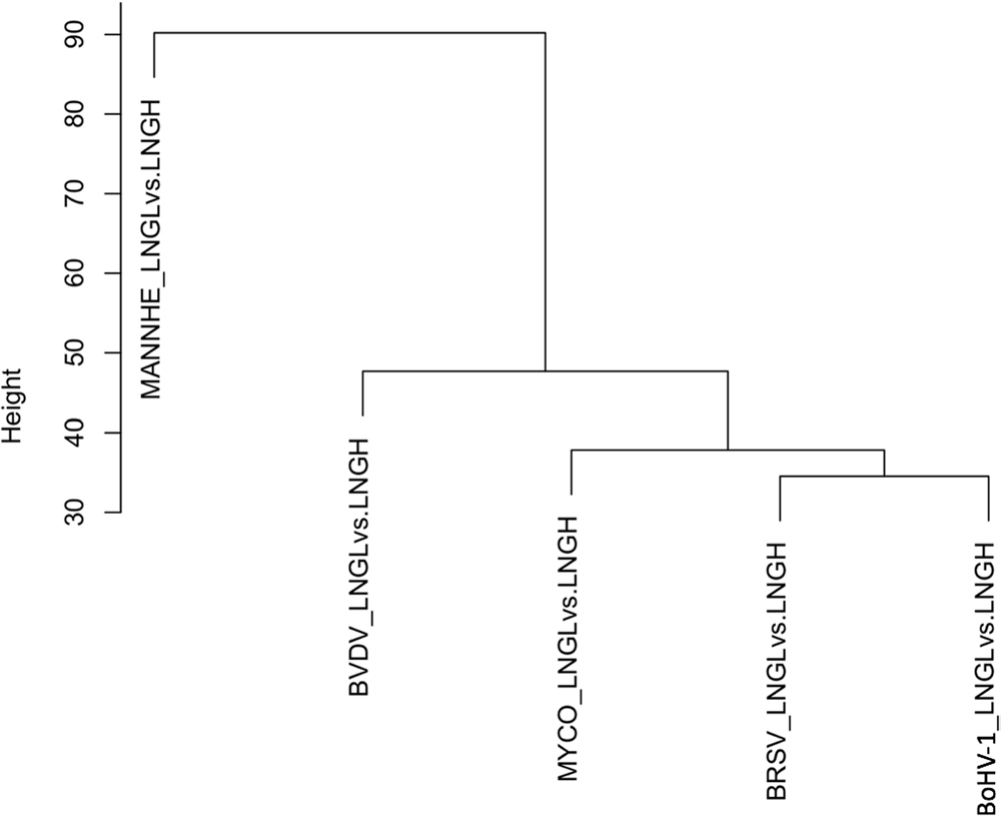
Hierarchical clustering of differentially expressed genes in lung lesion relative to healthy lung from the same individual following challenge with the different BRDC pathogens.

We next sought to understand how individual tissues respond to BRDC pathogens and whether they were associated with specific patterns of expression changes in response to challenge. To accomplish this, we first identified genes that were DE in a tissue-specific manner or that were ubiquitously DE in all five tissues (Table 2). Principal component analyses of the expression of genes DE in all tissues (marked as ‘ALL’**)** or specific tissues (marked using tissue abbreviations) are in Fig. 5A for each pathogen. The genes that were DE in all tissues tended to cluster differently for the different pathogens. We also conducted a principal component analysis of expression of DE genes between lung lesions and healthy lung tissue for each pathogen (Fig. 5B). These plots together show that gene expression patterns tend to cluster differently by host tissues and challenge pathogens. For example, infection by *M. haemolytica* induces more diverse transcriptional changes in lung lesion relative to healthy lung than any of the other pathogens (Fig. 6).

**Figure 7:**
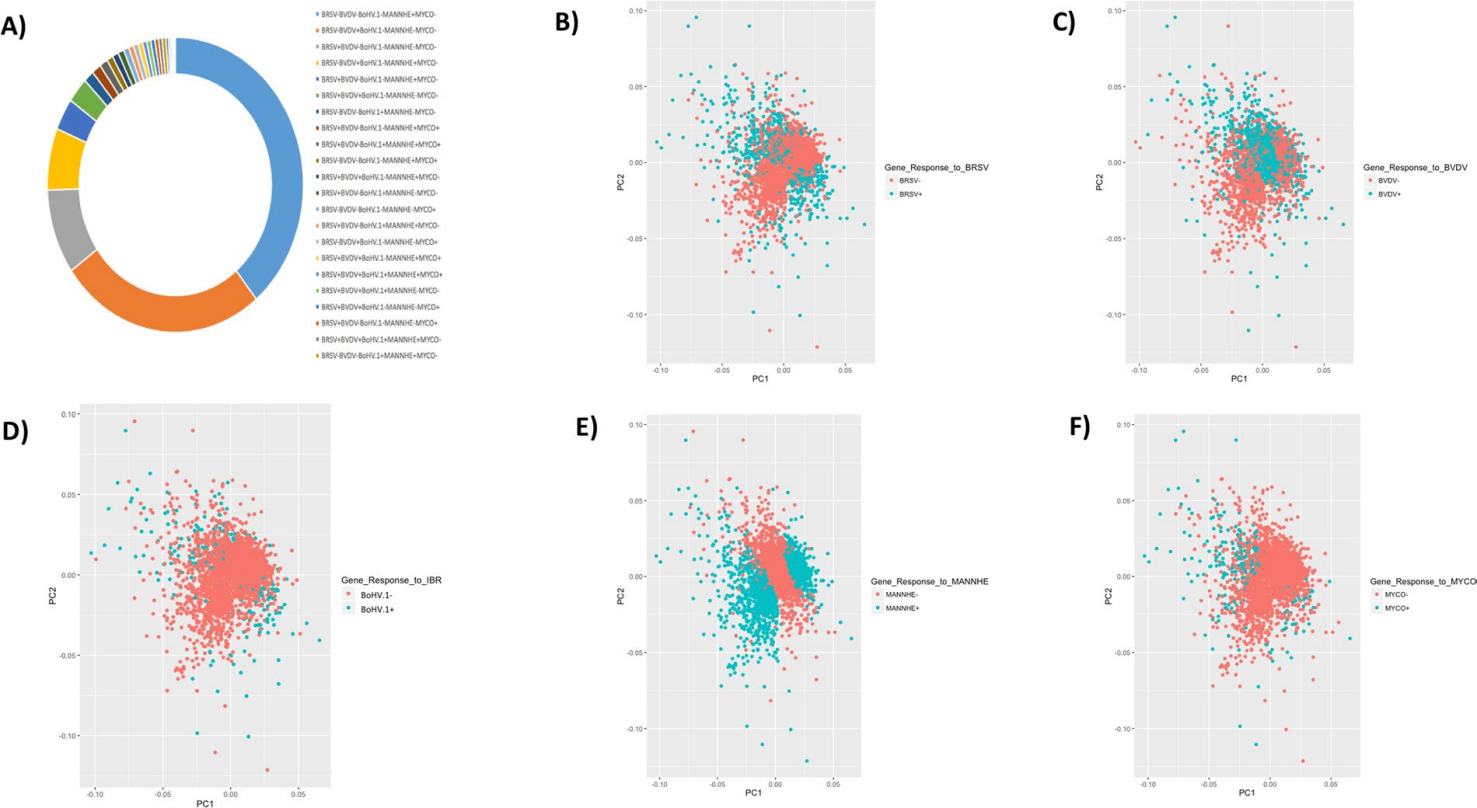
Patterns of differential gene expression in lung lesion relative to healthy lung. (**A**) Venn diagram showing the proportions of DE genes that share a host response to the different pathogens. The legend within panel A shows pathogen abbreviations where ‘+’ means the genes were differentially expressed in response to the pathogen and ‘−’ means gene expression differences were not significant for the pathogen. (**B–F**) Principal component analyses of gene expression levels for differentially expressed (blue) and non-differentially expressed (red) genes in response to challenge by the different pathogens.

In Fig. 7A, we show the relative proportions of genes that were DE between healthy lung and lung lesion for different combinations of BRDC pathogens. In particular, there were 1,256 genes DE in response to *M. haemolytica* but not in response to any of the other pathogens, 835 genes that were DE only in response to BVDV and 299 genes were DE in response only to BRSV. As a proportion of the total number of DE genes for each pathogen, the proportion for *M. haemolytica* (69.4%) was greater than that for BVDV (64.4%, *p* < 0.003) or for BRSV (39.7%, *p* < 0.00001). This suggests that the host immunological response in lung was predominantly associated with gene expression in response to the *M. haemolytica* challenge. Moreover, following *M. haemolytica* challenge, the expression levels for the DE and non-DE genes shown in the principal component analysis differed more for *M*. *haemolytica* than for all of the other BRDC pathogens (Fig. 7B–F), a result that was also supported by the clustering shown in Fig. 6. These data further support that the host transcriptional response in lung lesion differs substantially for *M. haemolytica* relative to the other BRDC pathogens.

### Tissue tropism is associated with differential gene networking

**Figure 8:**
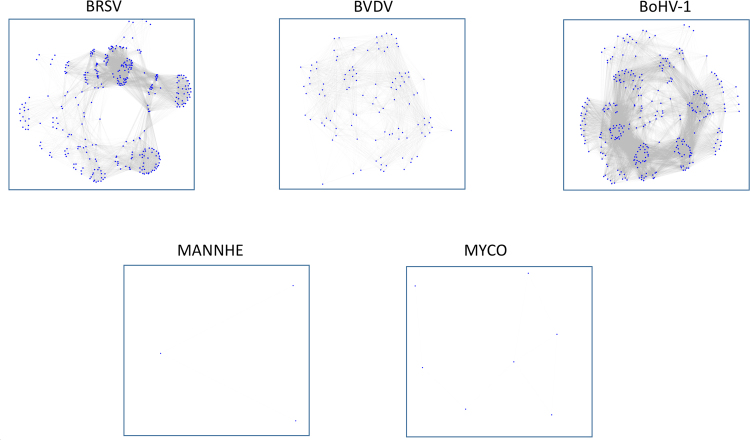
Lack of interaction among differentially expressed genes across tissues in response to experimental challenge by different BRDC pathogens. The nodes (genes) in each network are shown as blue dots and edges (interactions) are shown as grey lines. The pathogen abbreviations are shown above the gene networks corresponding to differentially expressed genes (across all tissues) in response to challenge by the individual pathogens.

While individual tissues appear to possess different roles in mounting a host response to infection, an interaction between tissue transcriptional responses may be necessary to mount an appropriate immune response to a specific pathogen. Accordingly, we hypothesized that the correlation between gene expression changes between tissues could be used to predict a tissue cooperative host response. As the number of DE genes varied considerably between the challenge pathogens (see Table 1), we randomly sampled 1,000 genes from the set of all DE genes across all tissues for each pathogen and generated mutual information matrices for each pathogen. From the mutual information analysis, we observed a strong deviation in mean mutual information for the viral challenges (BRSV = 0.2344, BVDV = 0.1238 and BoHV-1 = 0.1311) relative to the bacterial challenges (*M. haemolytica* = 0.0213 and *M. bovis* = 0.0204). This suggests that in response to the viral pathogens the different tissues have expression profiles that are mutually more informative to each other than are the tissue expression profiles in response to the bacterial challenges. Based on the weighted adjacency matrix generated from the pairwise mutual information data, the Maximum Relevance Minimum Redundancy expression networks (see Methods) revealed significantly different complexities for the viral and bacterial pathogen gene expression networks (Fig. 8).

### Networks and gene ontology for response genes

We annotated the functions of the DE genes for which the host response was tissue-specific or tissue-agnostic. For each pathogen, these two gene sets were analyzed for the over-representation of gene ontology (GO) terms using DAVID software. The results indicated that genes that were DE in multiple tissues were more likely to play roles in immune response and host defense mechanisms than were the genes that were DE in a tissue-specific manner (Table 3). Genes that were DE in all five tissues had functions in TLR, complement and coagulation cascades, endocytosis, chemokine and cytokine signaling, leukocyte transendothelial migration, cell adhesion and MAPK signaling. These events represent an orchestrated immune defense that occurs in all tissues to combat the infection. On the other hand, genes that were DE in a tissue-specific manner were more likely to be associated with GO terms such as extracellular region, contractile fiber part, myofibril, myosin filament and others. These genes included specific complement receptors regulating proinflammatory immune responses that are likely related to microenvironment immune responses and appear to define the nature of tissue tropism. For example, *CD70*, a member of Tumor Necrosis Factor ligand superfamily, was exclusively DE in the comparison of LNGL to control lung, as were genes encoding mitogen-activated protein kinases such as *MAP2K6*, *MAP2K3* and *MAPK8*. Moreover, surfactant genes encoding proteins secreted by type II alveolar macrophages that induce immune responses^13^ were also exclusively differentially regulated between LNGL and control lung.

**Table 3:** GO terms associated with genes that were differentially expressed in single (S) or multiple (M) tissues.

GO ID and Term	No. DE genes	*p*-value	Bonferroni corrected p-value	DE set ID*
GO:0005576~extracellular region	10	3.99E-04	0.023645061	BRSV-S
GO:0005615~extracellular space	6	7.69E-04	0.045113825	BRSV-S
GO:0005576~extracellular region	21	1.54E-07	1.05E-05	BRSV-M
GO:0048584~positive regulation of response to stimulus	7	8.01E-05	0.041798131	BRSV-M
GO:0006952~defense response	11	1.16E-06	6.20E-04	BRSV-M
GO:0048584~positive regulation of response to stimulus	7	8.01E-05	0.041798131	BRSV-M
GO:0032020~ISG15-protein conjugation	4	9.77E-07	5.21E-04	BRSV-M
GO:0005882~intermediate filament	6	1.01E-04	0.004546977	BDVD-M
GO:0045111~intermediate filament cytoskeleton	6	1.01E-04	0.004546977	BDVD-M
GO:0030855~epithelial cell differentiation	6	9.65E-07	1.49E-04	BDVD-M
GO:0060429~epithelium development	6	2.16E-05	0.003321463	BDVD-M
GO:0006955~immune response	28	3.15E-17	3.34E-14	BoHV-1-M
GO:0006954~inflammatory response	19	1.51E-16	1.18E-13	BoHV-1-M
GO:0009611~response to wounding	22	9.21E-16	9.41E-13	BoHV-1-M
GO:0006952~defense response	24	9.54E-16	1.06E-12	BoHV-1-M
GO:0002526~acute inflammatory response	10	1.42E-09	1.50E-06	BoHV-1-M
GO:0006955~immune response	28	3.15E-17	3.34E-14	BoHV-1-M
GO:0005615~extracellular space	25	2.23E-15	2.60E-13	BoHV-1-M
GO:0005576~extracellular region	41	2.97E-15	3.51E-13	BoHV-1-M
GO:0005125~cytokine activity	16	1.60E-13	4.48E-11	BoHV-1-M
GO:0044421~extracellular region part	28	1.71E-13	2.00E-11	BoHV-1-M
GO:0005125~cytokine activity	16	1.60E-13	4.48E-11	BoHV-1-M
GO:0006935~chemotaxis	10	1.01E-08	1.07E-05	BoHV-1-M
GO:0042330~taxis	10	1.01E-08	1.07E-05	BoHV-1-M
GO:0042379~chemokine receptor binding	8	1.08E-07	3.01E-05	BoHV-1-M
GO:0008009~chemokine activity	8	1.08E-07	3.01E-05	BoHV-1-M
GO:0007626~locomotory behavior	10	2.72E-06	0.00287541	BoHV-1-M
GO:0007610~behavior	12	4.48E-06	0.00474088	BoHV-1-M
GO:0002526~acute inflammatory response	10	1.42E-09	1.50E-06	BoHV-1-M
GO:0006953~acute-phase response	6	8.72E-07	9.24E-04	BoHV-1-M
GO:0006935~chemotaxis	10	1.01E-08	1.07E-05	BoHV-1-M
GO:0042330~taxis	10	1.01E-08	1.07E-05	BoHV-1-M
GO:0007626~locomotory behavior	10	2.72E-06	0.00287541	BoHV-1-M
GO:0009617~response to bacterium	9	9.28E-06	0.009792847	BoHV-1-M
GO:0030017~sarcomere	7	1.05E-08	1.03E-06	MANNHE-S
GO:0044449~contractile fiber part	7	1.90E-08	1.88E-06	MANNHE-S
GO:0030016~myofibril	7	2.87E-08	2.84E-06	MANNHE-S
GO:0043292~contractile fiber	7	4.75E-08	4.71E-06	MANNHE-S
GO:0032982~myosin filament	3	2.48E-04	0.024300011	MANNHE-S
GO:0008092~cytoskeletal protein binding	7	4.23E-04	0.047922373	MANNHE-S
GO:0030017~sarcomere	9	3.04E-11	3.22E-09	MYCO-S
GO:0044449~contractile fiber part	9	6.90E-11	7.31E-09	MYCO-S
GO:0030016~myofibril	9	1.21E-10	1.28E-08	MYCO-S
GO:0043292~contractile fiber	9	2.41E-10	2.56E-08	MYCO-S
GO:0031674~I band	7	3.38E-09	3.58E-07	MYCO-S
GO:0030018~Z disc	6	1.32E-07	1.40E-05	MYCO-S
GO:0030016~myofibril	6	9.84E-07	8.26E-05	MYCO-M
GO:0043292~contractile fiber	6	1.49E-06	1.25E-04	MYCO-M
GO:0016459~myosin complex	5	3.91E-05	0.003277612	MYCO-M
GO:0032982~myosin filament	3	2.31E-04	0.01925287	MYCO-M
GO:0030017~sarcomere	4	5.20E-04	0.042770803	MYCO-M
GO:0005615~extracellular space	8	6.65E-05	0.00557453	MYCO-M

*DE set ID represents genes that were differentially expressed in response to a pathogen (BRSV, BVDV, BoHV-1, MANNHE or MYCO) either in a single tissue (designated as –S) or in multiple tissues (designated as –M).

The comparison of GO terms for the tissue-specific or tissue-agnostic DE genes in response to bacterial or viral challenges is shown in Supplementary Figure 1. This figure shows that tissue-specific or tissue-agnostic host transcriptional response GO terms tend not to overlap between the bacterial and viral challenges and when they were in common, tended to be for the bacterial pathogens.

### Identification of key immune genes

We identified immune function-related genes by querying the DE genes to the innate immunity database InateDB (http://www.innatedb.com) for *Bos taurus* (Supplementary Table 2). The immune function related genes that were DE between lung lesion and apparently healthy lesion-free lung tissues are listed in Supplementary Table 3. The Maximum Relevance Minimum Redundancy network analysis of expression changes for these immune function-related genes revealed an extensive interaction between tissues in response to the challenges (e.g., for BoHV-1 shown in Supplementary Figure 2). Using degree centrality statistics to predict the key players within the gene expression networks, we predicted the top three key players among the immune function gene networks in response to each of the challenge pathogens (Table 4). The predicted key players have major roles in the defense against bacterial and viral infections. For example, *BPIFA1* is expressed in the upper airways and nasopharyngeal regions in human and encodes an antimicrobial protein with antibacterial activity^14^. Several of the predicted key players are members of the C1Q ‘complement’ system gene family and are involved in host-pathogen interactions including respiratory tract inflammations^15^. *APCS* encodes amyloid P component, serum that is associated with laryngeal amyloidosis in humans^16^, and *AKIRIN2* has been described as a ‘novel player’ in the transcriptional control of innate immunity^17^.

**Table 4:** Predicted top 3 key players in the immune function gene networks in response to challenge by BRDC pathogens. Prediction was based on degree centrality estimation within each of the mutual information networks of differentially expressed immune function genes. The degree centrality score is a value that represents how well the model predicts the three genes to occupy the central nodes in the network.

Pathogen	Key players	Centrality score
BRSV	*BPIFA1, C1QB, C1QBP*	1.34
BVDV	*AKIRIN2, APCS, BPIFA1*	1.84
BoHV-1	*APCS, C1QA, C1QB*	2.08
MANNHE	*C1QA, C1QB, C4BPA*	2.85
MYCO	*BPIFA1, C9, CD14*	0.56

### Pathway prediction of differentially expressed immunity genes

**Figure 9:**
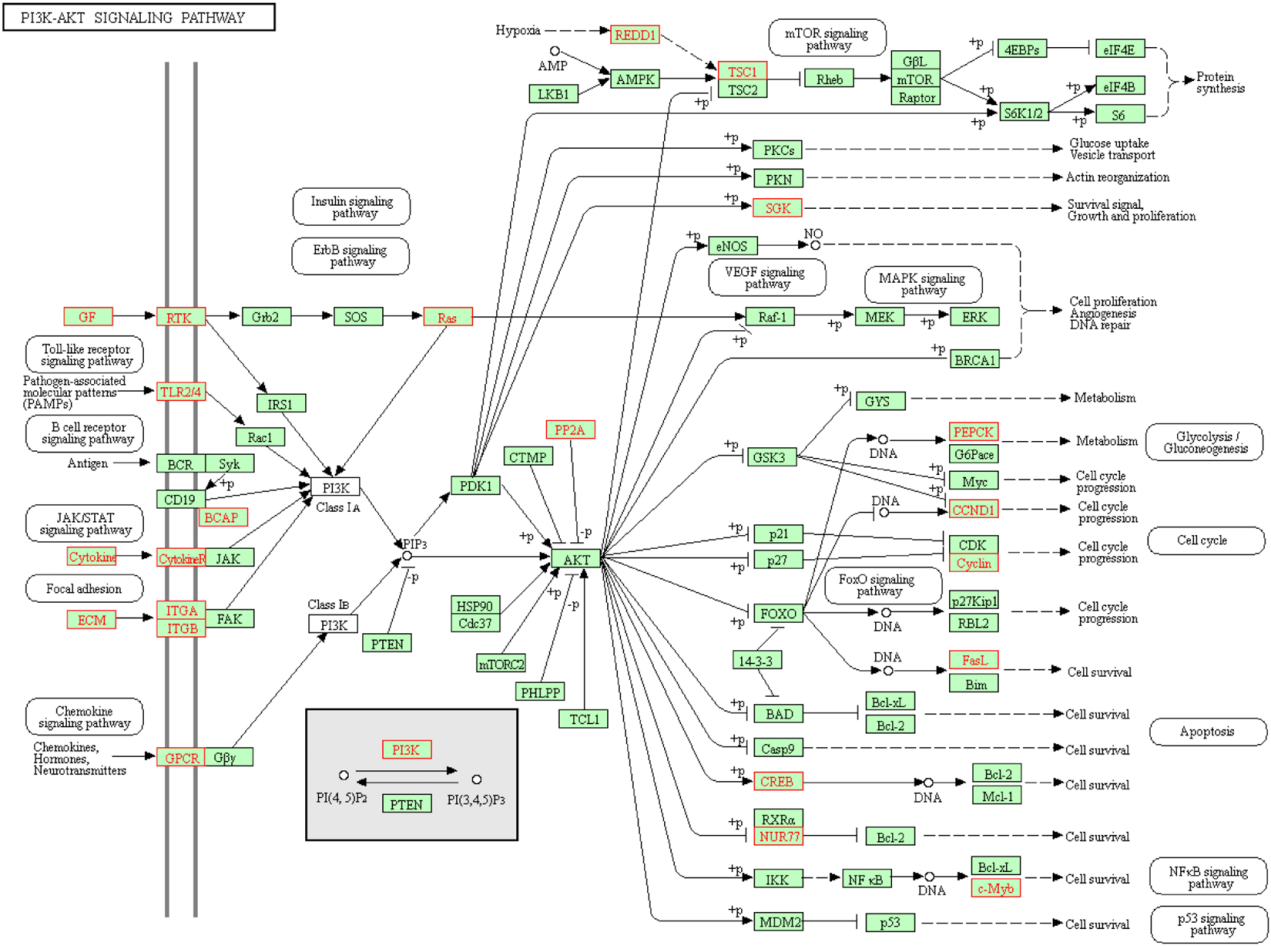
KEGG (Kanehisa & Goto, 2000) PI3K-Akt signaling pathway (map04151) is enriched for genes differentially expressed between lesion and healthy lung tissue in cattle challenged with BRDC pathogens. The genes shown in red are differentially expressed and up-regulated in lung lesions relative to healthy lung tissue.

Finally, we sought to establish which pathways might be involved in host transcriptional responses to BRDC pathogens. The genes that were found to be responsive across tissues were tested to determine if any *Bos taurus* KEGG^18^ pathway was significantly over-represented by DE genes. We found that several disease-associated pathways, including those involved in host response to viral infections, were significantly associated with the genes that were consistently DE across all tissues (Table 5). We also observed the RIG-I-like receptor, NF-kappa B and NOD-like receptor signaling pathways to be significantly enriched for DE genes suggesting that these pathways play roles in a tissue cooperative host response to infection. Phagosome and complement and coagulation cascades were also significantly enriched for DE genes further suggesting their roles in response to early infection events that may be indicative of infection spreading from one tissue to another. In lung tissues, we analyzed lesion-associated DE genes for KEGG pathway enrichment, and found that pathways such as the ECM-receptor interaction, focal adhesion, PI3K-Akt signaling pathway, along with other specific metabolic pathways were significantly enriched (Table 6). The greatest number of DE genes was associated with the PI3K-Akt pathway, and this pathway is upstream of important molecular cascades known to be associated with cell cycle, programmed cell death and p53 signaling (Fig. 9). In Fig. 9, we observe specific DE genes (shown in red text) such as *PEPCK*, *CCND1*, *FASLG* and *MYB* that are directly upstream of these events (cell cycle, apoptosis and p53 signaling). This suggests that these DE genes within the PI3K-Akt pathway may play important roles in eliciting downstream cascades in lung lesions.

**Table 5:** List of pathways predicted to be involved in tissue tropism of host gene expression changes in response to challenge by BRDC pathogens. Pathways are enriched for genes that were DE in all analyzed tissues.

KEGG Pathway	No. DE Genes	Fold Enrichment	*p*-value
bta05164:Influenza A	13	5.70	2.24E-06
bta05144:Malaria	7	9.72	7.03E-05
bta05168:Herpes simplex infection	11	4.39	1.73E-04
bta05160:Hepatitis C	9	5.13	3.25E-04
bta05134:Legionellosis	6	7.75	9.60E-04
bta05162:Measles	8	4.33	0.002279078
bta04622:RIG-I-like receptor signaling	6	5.99	0.003044239
bta05133:Pertussis	6	5.91	0.003222503
bta05132:Salmonella infection	6	5.32	0.005056302
bta05150:Staphylococcus aureus infection	5	6.43	0.007185489
bta04064:NF-kappa B signalin	6	4.85	0.007444893
bta05323:Rheumatoid arthritis	6	4.65	0.008894029
bta05161:Hepatitis B	7	3.59	0.012652915
bta04610:Complement and coagulation cascades	5	5.12	0.015668557
bta04145:Phagosome	7	3.20	0.021102066
bta05322:Systemic lupus erythematosus	7	2.98	0.028594761
bta04621:NOD-like receptor signaling	4	5.66	0.032465602
bta05152:Tuberculosis	7	2.84	0.034817614

**Table 6:** Pathways associated with genes differentially expressed between lesion and healthy lung tissue.

Pathway	Count	Fold Enrichment	*p*-Value
bta04512:ECM-receptor interaction	31	2.87	6.86E-08
bta04510:Focal adhesion	50	1.94	4.51E-06
bta04151:PI3K-Akt signaling pathway	72	1.67	8.80E-06
bta00010:Glycolysis/Gluconeogenesis	21	2.69	4.20E-05
bta01130:Biosynthesis of antibiotics	42	1.64	0.001235618
bta01200:Carbon metabolism	21	1.55	0.044739966
bta05410:Hypertrophic cardiomyopathy (HCM)	20	2.02	0.003437553
bta05414:Dilated cardiomyopathy	21	1.97	0.003534447
bta05412:Arrhythmogenic right ventricular cardiomyopathy (ARVC)	17	1.99	0.008840512
bta00360:Phenylalanine metabolism	7	2.69	0.0373829
bta04925:Aldosterone synthesis and secretion	17	1.74	0.030708624

## Discussion

We performed a detailed analysis of tissue transcriptional responses to understand the involvement of lymphoid and lung tissues in the normal immune response to infection by BRDC pathogens at the peak of clinical signs of disease. Relative to spontaneous infections, the intranasal inoculations for viruses and intra-tracheal inoculations for bacteria may have affected pathogen spreading and the localization and extent of the lesions as well as potentially the extent of lymphoid tissue involvement. While we cannot exclude this possibility, the respiratory viruses are known to be infective by the inhalation route and we considered the tracheal administration of the bacterial pathogens to best represent the normal mode of lung infection and using these routes of inoculation we successfully induced clinical signs and lung pathology in the beef steers following each of the single pathogen experimental challenges^9^. Consequently, we also expect that tissues analyzed produced appropriate genetic responses to each of the pathogens.

Respiratory epithelial cells can sense viral pathogens through diverse molecules such as TLR3, TLR7, TLR9, RIGI, MDA5 and NLRP3, leading to the activation of type I interferon and the induction of a range of antiviral mechanisms^11,19,20^. While we detected the up-regulation of IFNγ, we detected no type I interferon response in the animals challenged with BVDV. A recent study has shown that BVDV infection induces IFNγ secretion during acute phase signaling and that lymphoid tissues serve both as possible sources of IFNγ and as target organs for its effects^21^. Production of nitric oxide and reactive oxygen species were consistently predicted to be activated in the tissues of the BRSV challenged animals. The up-regulation of nitritic oxide and reactive oxygen species either reflects the induction of these species by the virus to facilitate replication^22^, or are hallmarks of oxidative stress and the induction of apoptosis^23^. We also detected the differential regulation of cytokine production by *IL17A* and *IL17F* which down-regulate *IL10*, *IL6* and *CCL3* in LNGL. Furthermore, we detected the suppression of leukocyte extravasation signaling in PGT and the up-regulation of thrombin signaling in all of the tissues. Non-cytopathic BVDV strains such as strain 890 used for inoculation in this study are known to suppress proinflammatory cytokines and co-stimulatory molecules^24^.

The bovine tracheal antimicrobial peptide was found to be DE in all tissues except NLN. While this gene was predominantly up-regulated, it was found to be down-regulated in NLN for animals challenged with BoHV-1 and *M. haemolytica*. However, pathogen produced toxins are known to have the ability to repress host defensin transcription as a mechanism to combat the host immune response^25^. Consistent with the role of reactive oxygen species in the first line of defense against pathogens, genes with functions involving reactive oxygen species were also found to be DE. Reactive oxygen species produced by phagocytes may help in the removal of pathogens that are resistant to antimicrobial peptides; however, potentially at the expense of tissue damage. We appear to have captured most of the events that caused lung tissue damage. The leukotriene biosynthesis, γ-glutamyl cycle and eicosanoid signaling pathways were inferred as being the most strongly induced pathways in the lung lesions of the BVDV challenged animals. Genes encoding γ-glutamyl transpeptidases, which are present in these pathways, are known to cleave leukotriene^26^ and examination of the genes induced in this pathway revealed that they were involved in converting leukotriene-D4 to leukotriene-E4. Leukotrienes C4, D4 and E4 are released from lung tissue exposed to allergens and are involved in immediate hypersensitivity reactions^27^. The leukotriene biosynthesis pathway was also induced by *M. haemolytica* infection, however, *ALOX5* was identified as the up-regulated gene responsible for the conversion of leukotriene A4 into other leukotrienes in the tissues of animals from this challenge group.

We previously analyzed pathogen-specific host responses in the bronchial lymph nodes of these steers^9^. Consistent with our current findings, the common pathways underlying response to several of the challenge organisms included pathways with major roles in innate immunity that are not tissue or pathogen-specific. However, we also identified several pathways that were enriched for DE genes in all analyzed tissues including BLN, such as Toll-like receptor signaling, chemokine signaling and granulocyte adhesion and diapedesis, as well as conserved predicted upstream regulators including *IL1B* thus supporting their roles in the immune response to the pathogens of the BRDC. Despite the finding of common pathways enriched for DE genes across challenge pathogens, different genes appear to be responsible for pathway activation. Whether this result reflects tissue-specific activation of pathways or a lack of power of single tissue analyses with four biological replicates is not clear. The majority of the DE genes identified in the current study were associated with functions in complement and coagulation cascades, endocytosis, chemokine and cytokine signaling, leukocyte transendothelial migration, cell adhesion and MAPK signaling. These events represent an orchestrated immune defense that occurs in all tissues to combat the infection. We observed numerous genes exclusively DE in the PGT that operate within pathways related to the ribosome and spliceosome as well as the post-transcriptional modification of RNA. In particular, we detected genes related to EIF2 signaling and the regulation of the eIF4 and p70S6K signaling pathways. Similarly, the genes that were exclusively DE in the RLN were related to transcriptional regulation pathways. While these responses to the viral infections were also observed in other tissues, an increase in the number of DE genes related to signaling functions was observed in both the PGT and RLN.

Besides identifying genes with expression changes in response to different BRDC pathogens, our study shows that the host response to respiratory diseases is modulated by the nature of cross-talk between response genes in different lymphoid tissues. The network of response genes is modulated by key immune function related genes that we predicted using ‘key player’ analysis. While key player analysis has previously been applied primarily to the study of social networks, our implementation in the analysis of gene expression networks provided a useful approach for characterizing the nature of immune function genes in BRD. Our results further show that tissue gene expression profiles in response to infection by BRDC pathogens can generally be clustered based on tissues rather than pathogens (Fig. 1) suggesting that different lymphoid tissues play signature roles in mounting the host’s response to respiratory infections. This is necessary to trigger appropriate host immunity to defend against pathogen infections where clinical manifestation varies depending on strain of virus, host, immunity and other factors^28,29^. We show in this work that tissue cooperative expression changes in genes differ significantly between viral and bacterial infections. This suggests that tissue tropism of the host’s transcriptional response is dependent upon the invading pathogen and may be dynamic to counter the evolution of pathogen virulence. Host-pathogen interactions are dependent upon the binding receptors of appropriate cells in the target tissues and tropism in host tissue gene expression may be a key determinant of how well a pathogen can successfully spread its infection from one tissue to another.

## Materials and Methods

### Animal ethics statement

This study was conducted under animal use protocol #16424 approved by the Institutional Animal Care and Use Committee of the University of California at Davis. The protocol adheres to the Federation of Animal Science Societies Guide for the Care and Use of Agricultural Animals in Research and Teaching.

### Animal sampling and challenge

Crossbred steers were generated at the University of California Davis Sierra Field Station located in Brown’s Valley, California by mating Angus bulls to advanced generation Angus-Hereford crossbred dams. Blood from steers that had not been vaccinated against any pathogen from the BRDC was tested for antibodies against each bacterial and viral pathogen and those found to be seronegative, or to have the lowest titers against each of the challenge pathogens, were selected at 6–8 months of age for the challenge experiment that was conducted at the University of California Davis. The steers were provided water *ad libitum* while maintained in pens and a fed a 65% concentrate starter diet. Pathogen challenges were performed sequentially on pen-grouped animals under strict biosecurity protocols. A period between challenges with different pathogens was used to prevent cross-infection^12^.

A pilot project was conducted during the summer of 2011 to determine the optimum pathogen doses to administer to the challenged animals and also the timing of clinical signs of peak infection to determine the euthanasia end-point for each challenge group. In the optimized challenge experiment conducted in summer of 2012, groups of N = 4 animals were individually challenged with BRSV, BVDV or BoHV-1 via a nebulizer or with *M. haemolytica* or *M. bovis* administered by an intratracheal tube inserted through the ventral nasal meatus until the end was approximately in the mid-trachea. Optimized pathogen doses per animal were: BRSV (1.6 × 10^5^/ml × 8.5 ml), BVDV (2.0 × 10^8^/ml × 8.5 ml), BoHV-1 (1.0 × 10^7^/ml × 8.5 ml), *M. haemolytica* (4.8 × 10^11^ CFU) and *M. bovis* (7.0 × 10^10^ CFU). Using a nebulizer, two of the control animals were aerosol administered with 8.5 ml of tissue culture media while a second pair of controls were inoculated with 8.5 ml of phosphate buffered saline. The intratracheal inoculation of phosphate buffered saline was followed by sufficient air to remove all saline from the tube. The aerosol administration of tissue culture media was designed to mimic one of the mechanisms of viral exposure. Strains were previously reported^12^.

Clinical scores were recorded daily on each animal from 3 days prior to challenge until the day of euthanasia using an index that was a combination of observations. None of the animals required analgesic or anesthetic treatment except topical lidocaine, which was applied to the nasal passages prior to the insertion of the intratracheal tube that was used to administer each challenge pathogen. Clinical signs peaked on days 7, 15, 6, 5, and 15 post-challenge for BRSV, BVDV, BoHV-1, *M. haemolytica*, and *M. bovis*, respectively, at which point the steers from each group were euthanized. These were not considered to be humane end-points. A certified veterinary pathologist removed tissues for examination and the samples collected for transcriptome analysis were immediately frozen in liquid nitrogen. Tissues remained frozen at −80 °C until RNA was isolated for analysis. One animal that was challenged with *M. bovis* was euthanized early and tissues were not collected. This left 23 animals from which samples of each tissue were harvested from control animals (N = 4) and those animals artificially challenged with BRSV (N = 4), BVDV (N = 4), BoHV-1 (N = 4), *M. haemolytica* (N = 4) and *M. bovis* (N = 3) and were analyzed by RNA-Seq.

### RNA isolation and sequencing

RNA was extracted from a total of 50–100 mg of frozen tissue using RNeasy Plus Universal Mini Kits (Qiagen, Hilden, Germany). Briefly, 900 μl of QIAzol (similar to acidic buffered phenol) was added to the frozen tissue sample, which was immediately homogenized and incubated at room temperature for 5 min for the dissociation of the nucleoprotein complex, and then 100 μl of genomic DNA eliminator solution and 180 μl of chloroform were added. Samples were centrifuged at 12000 g for 15 min at 4 °C and after transferring the aqueous layer to a fresh tube, 600 μl of 100% ethanol was added and mixed by pipette. The mixture was transferred to an RNeasy spin column (based on silica-membrane technology) and centrifuged at 8000 g for 15 sec at room temperature and was then washed with 700 μl of Qiagen RWT buffer and 500 μl of Qiagen RPE buffer. The RNA was eluted with 100 μl RNase-free water and was placed at 4 °C. RNA purity and concentration were evaluated using a NanoDrop 1000 v1.3.2 (Thermo Fisher Scientific, Waltham, MA) and Qubit 3.0 Fluorometer (Thermo Fisher Scientific). The extent of RNA degradation was initially assessed by the electrophoresis of 1 μg of RNA on a 1.0% agarose gel. Finally, RNA quality was assessed for each sample using a Fragment Analyzer^TM^ (Advanced Analytical Technologies, Inc, Ames, IA) with a standard sensitivity RNA analysis kit and we accepted samples with an RNA quality number of at least 8.0 for library construction.

A total of 10 μg of RNA from each tissue was processed using the TruSeq RNA Sample Preparation Kit (Illumina, San Diego, CA) to prepare samples for sequencing. Oligo dT magnetic beads were used to purify polyadenylated RNA from the total RNA which was then fragmented with divalent cations under elevated temperature. First strand cDNA was synthesized using random hexamer primers and then the second strand was synthesized. The double-stranded cDNA was end-repaired and the 3′ ends adenylated. Finally, to produce the sequencing library, universal adapters were ligated to the cDNA fragments that were then amplified by solid phase polymerase chain reaction. Each library was evaluated using a Fragment Analyzer and equimolar amounts were used to create 7 library pools, which were each sequenced (2 × 50 bp) on a single lane of a HiSeq. 2000. We produced an average of 50,778,115 reads per sample.

### Processing of sequence reads

Adapter sequences were trimmed from the sequence reads as previously described^30^.

### Read alignment

Computation for this work was performed on the high performance computing infrastructure provided by Research Computing Support Services at the University of Missouri, Columbia MO. TopHat v2.0.6^31^ was used to map the trimmed sequence reads to the NCBI *Bos taurus* virtual transcriptome build and the UMD3.1 reference assembly. TopHat first used Bowtie to align the sequence reads to the virtual transcriptome build. Reads that failed to map to the virtual transcriptome build were next mapped to the UMD3.1 reference assembly. Reads that mapped to UMD3.1 were converted to genomic mappings, spliced if required, and then merged with the transcriptome mapped reads. No more than 2 mismatches were allowed in the alignment of each read pair and the remaining TopHat parameters were left at default values.

### Testing for differential expression

Cuffdiff^32^ was used to estimate transcript abundance levels as Fragments Per Kilobase of exon per Million fragments mapped for each gene in each challenge group and to test transcripts for differential expression against controls. The *p*-values for each performed test were transformed to q-values using the Benjamini-Hochberg correction^33^ to correct for multiple testing.

### Cluster analysis, network inference and key player analysis

Expression data for all genes were subjected to hierarchical clustering among samples to generate a dendrogram. The Euclidean distance measure was used to calculate distance and data fitting was performed using the method of Ward^34^. The model-based cluster analysis was performed using the *R* ‘mclust’ package using a finite normal mixture modeling method^34^. The Bayesian Information Criterion, implemented in mclust, was chosen for model selection to cluster the genes based on their expression data Eigenvalue Decomposition Discriminant Analysis (EDDA) method^35^. To infer the extent of interactions between genes within a cluster, network analysis was performed using ‘minet’^36^ based on mutual information values that were calculated using the Spearman correlation estimator. The square symmetric matrix containing all mutual information values for all genes was used to generate a weighted adjacency matrix by the ‘maximum relevance minimum redundancy’ method^37^. The adjacency matrix was then used to identify and plot the co-expression networks. Key genes in each network were predicted from degree centrality scores (that predict how central the genes are relative to the overall network structure) using a ‘key player’ analysis approach, a method used in analyzing social networks^38^.

### Statistical tests

Chi-Square contingency table tests were performed using counts of numbers of DE genes for single and multi-tissue expression to examine associations between host tissue and BRDC pathogen. All of the principal component analyses and data visualization were performed using *R*. For the randomization test of DE genes in the network analysis, we selected a fixed number of genes (n = 1000) to create the expression networks. We repeated the process of sampling 1000 genes and calculating pairwise mutual information scores and used the average mutual information scores across replicates to perform Chi-Square tests to examine associations between pathogen and tissue combinations.

### Annotation of differentially expressed genes

The Ingenuity Pathway Analysis software (http://www.ingenuity.com) was used to understand the biological processes regulated by the DE genes. The gene ontology and KEGG pathway analyses of DE genes were performed using DAVID Bioinformatics Resources 6.8 (https://david.ncifcrf.gov). Innate immune genes of cattle curated at InnateDB (http://www.innatedb.com) were used to identify immune function genes found to be DE in the RNA-Seq data. Pathway painting was performed using KEGG mapper (http://www.genome.jp/kegg/mapper.html).

### Accession Codes

Sequence data have been submitted to the NCBI Sequence Read Archive under BioProject PRJNA272725. Supplementary Table 1 contains sample identification information for each of the experimentally challenged animals and tissues.

## Electronic supplementary material

Supplementary Information

Supplementary DataSet

## References

[1] Edwards TA (2010). Control methods for bovine respiratory disease for feedlot cattle. Vet. Clin. North Am. Food Anim. Pract.

[2] Kirchhoff J, Uhlenbruck S, Goris K, Keil GM, Herrler G (2014). Three viruses of the bovine respiratory disease complex apply different strategies to initiate infection. Vet Res.

[3] Rice JA, Carrasco-Medina L, Hodgins DC, Shewen PE (2007). Mannheimia haemolytica and bovine respiratory disease. Anim Health Res Rev.

[4] Hodgins, D. C., Conlon, J. A. & Shewen, P. E. Respiratory viruses and bacteria in cattle in *Polymicrobial Diseases* (Ed. Brogden, K. A. & Guthmiller, J. M.) 12 (ASM Press, 2002). 21735561

[5] Gagea MI (2006). Diseases and pathogens associated with mortality in Ontario beef feedlots. J Vet Diagn Invest.

[6] Taylor JD, Fulton RW, Lehenbauer TW, Step DL, Confer AW (2010). The epidemiology of bovine respiratory disease: What is the evidence for predisposing factors?. Can Vet J.

[7] Westermann AJ, Gorski SA, Vogel J (2012). Dual RNA-Seq of pathogen and host. Nat Rev Microbiol.

[8] Marioni JC, Mason CE, Mane SM, Stephens M, Gilad Y (2008). RNA-Seq: an assessment of technical reproducibility and comparison with gene expression arrays. Genome Res.

[9] Tizioto PC (2015). Immunological response to single pathogen challenge with agents of the Bovine Respiratory Disease Complex: An RNA-sequence analysis of the bronchial lymph node transcriptome. PLoS One.

[10] McCall LI, Siqueira-Neto JL, McKerrow JH (2016). Location, Location, Location: Five Facts about Tissue Tropism and Pathogenesis. PLoS Pathog.

[11] Caswell JL (2014). Failure of respiratory defenses in the pathogenesis of bacterial pneumonia of cattle. Vet Pathol.

[12] Gershwin LJ (2015). Single pathogen challenge with agents of the bovine respiratory disease complex. PLoS One.

[13] van Iwaarden F, Welmers B, Verhoef J, Haagsman HP, van Golde LM (1990). Pulmonary surfactant protein A enhances the host-defense mechanism of rat alveolar macrophages. Am. J. Respir. Cell Mol Biol.

[14] Britto CJ, Cohn L (2015). Bactericidal/permeability-increasing protein fold-containing family member A1 in airway host protection and respiratory disease. Am J Respir Cell Mol Biol.

[15] Agarwal V, Blom AM (2015). Roles of complement C1q in pneumococcus-host interactions. Crit Rev Immunol.

[16] Lewis JE, Olsen KD, Kurtin PJ, Kyle RA (1992). Laryngeal amyloidosis: a clinicopathologic and immunohistochemical review. Otolaryngol Head Neck Surg.

[17] Tartey S, Takeuchi O (2015). Chromatin remodeling and transcriptional control in innate immunity: emergence of Akirin2 as a novel player. Biomolecules.

[18] Kanehisa M, Goto S (2000). KEGG: Kyoto Encyclopedia of Genes and Genomes. Nucleic Acids Res.

[19] Sadler AJ, Williams BR (2008). Interferon-inducible antiviral effectors. Nat Rev Immunol.

[20] Shahangian A (2009). Type I IFNs mediate development of postinfluenza bacterial pneumonia in mice. J Clin Invest.

[21] Smirnova NP (2014). Induction of interferon-gamma and downstream pathways during establishment of fetal persistent infection with bovine viral diarrhea virus. Virus Res.

[22] Peterhans E (1997). Reactive oxygen species and nitric oxide in viral diseases. Bio Trace Elem Res.

[23] Schweizer M, Peterhans E (1999). Oxidative stress in cells infected with bovine viral diarrhoea virus: a crucial step in the induction of apoptosis. J Gen Virol.

[24] Lee SR, Pharr GT, Boyd BL, Pinchuk LM (2008). Bovine viral diarrhea viruses modulate toll-like receptors, cytokines and co-stimulatory molecules genes expression in bovine peripheral blood monocytes. Comp Immunol Microbiol Infect Dis.

[25] Chakraborty K (2008). Bacterial exotoxins downregulate cathelicidin (hCAP-18/LL-37) and human beta-defensin 1 (HBD-1) expression in the intestinal epithelial cells. Cell Microbiol.

[26] Mayatepek E (2004). Synthesis and metabolism of leukotrienes in gamma-glutamyl transpeptidase deficiency. J Lipid Res.

[27] Samuelsson B (1983). Leukotrienes: mediators of immediate hypersensitivity reactions and inflammation. Science.

[28] Ridpath JF (2010). Bovine viral diarrhea virus: global status. Vet Clin North Am Food Anim Pract.

[29] Ridpath JF (2013). Immunology of BVDV vaccines. Biologicals.

[30] Chapple RH (2013). Characterization of the rat developmental liver transcriptome. Physiol Genomics.

[31] Trapnell C, Pachter L, Salzberg SL (2009). TopHat: Discovering splice junctions with RNA-Seq. Bioinformatics.

[32] Trapnell C (2012). Differential gene and transcript expression analysis of RNA-Seq experiments with TopHat and Cufflinks. Nat Protoc.

[33] Trapnell C (2013). Differential analysis of gene regulation at transcript resolution with RNA-Seq. Nat Biotechnol.

[34] Ward JH (1963). Hierarchical grouping to optimize an objective function. J Am Stat Assoc.

[35] Fraley C, Raftery AE (2002). Model-based clustering, discriminant analysis, and density estimation. J Am Stat Assoc.

[36] Meyer PE, Lafitte F, Bontempi G (2008). Minet: A R/Bioconductor package for inferring large transcriptional networks using mutual information. BMC Bioinformatics.

[37] Ding C, Peng H (2005). Minimum redundancy feature selection from microarray gene expression data. J Bioinform Comput Biol.

[38] Borgatti SP (2006). Identifying sets of key players in a social network. Comput Math Organiz Theor.

