# Supplementary material for: Tissue Tropism in Host Transcriptional Response to Members of the Bovine Respiratory Disease Complex
Source: Sci Rep. 2017 Dec 20;7:17938. doi: 10.1038/s41598-017-18205-0 (PMC5738336; doi:10.1038/s41598-017-18205-0)
Supplement: Supplementary file 1 — Supplementary Information [file 41598_2017_18205_MOESM1_ESM.pdf]

Tissue Tropism in Host Transcriptional Response to Members of the Bovine Respiratory Disease Complex

Susanta K. Behura, Polyana C. Tizioto, JaeWoo Kim, Natalia V. Grupioni, Christopher M. Seabury, Robert D. Schnabel, Laurel J. Gershwin, Alison L. Van Eenennaam, Rachel Toaff-Rosenstein, Holly L. Neibergs, Luciana C. Regitano & Jeremy F. Taylor

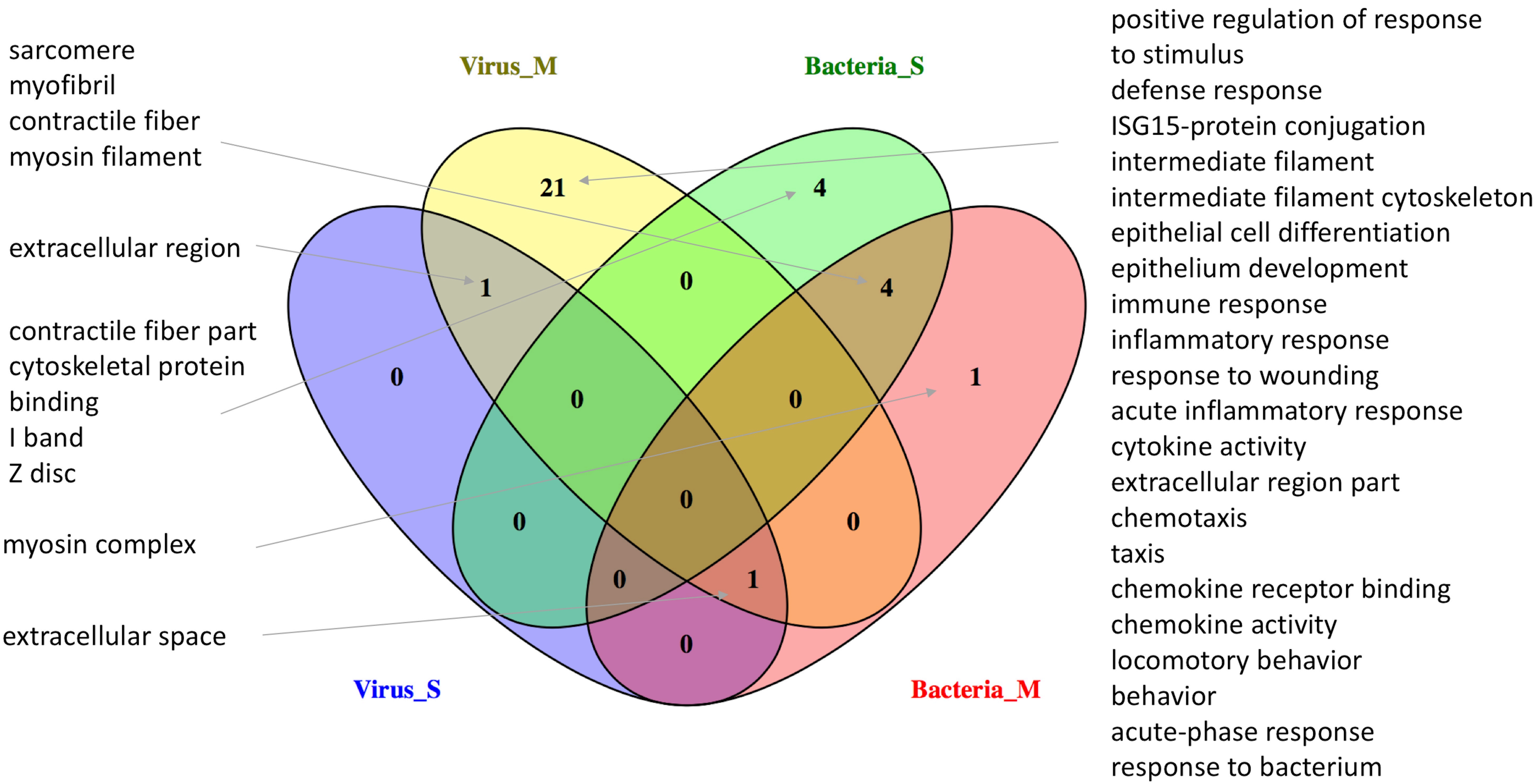

Supplementary Figure 1. Venn diagram showing the number of significant GO terms that were common or specific to differentially expressed genes in single (_S) or multiple (_M) tissues in response to bacterial or viral challenges.

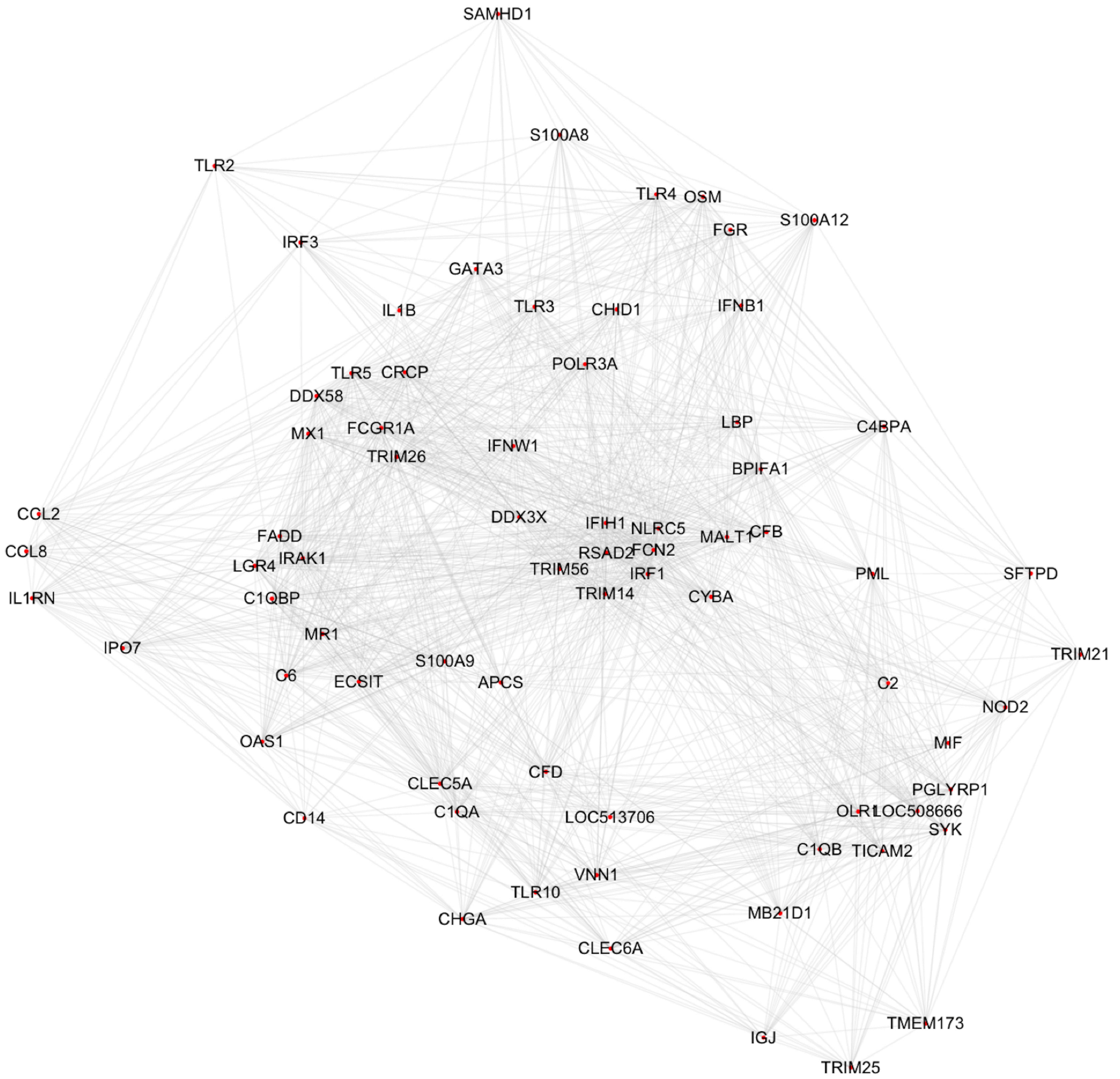

Supplementary Figure 2. Expression network for immune function-related genes differentially expressed in multiple tissues following challenge with BoHV-1.

Table S1. Sample identifier, sequence read statistics and accession numbers for Small Read Archive (SRA) submissions. See abbreviations below the table.

Lab_ID	Tissue_ID	Tissue	Acession Number	N.Reads	N.Reads(F+R)	Left_mapped	Left_MA	right_mapped	right_MA	Aligned_pairs	MA	discordant_pair	overal_mapp.	concordant_pair
86684	52239	LNGH	SRX1254108	46390492	92780984	42670520	1744946	41395822	1683148	39763671	1578292	992287	0.906	0.836
86685	52260	LNGH	SRX1254113	49114190	98228380	45063486	1865397	43723052	1797348	42010589	1687852	1057303	0.904	0.834
86686	52281	LNGH	SRX1254118	53777154	107554308	49224627	2111922	48328029	2067170	46295413	1936473	1109892	0.907	0.840
86687	52302	LNGH	SRX1254123	47190948	94381896	43136549	1832840	41923918	1770298	40242173	1669909	1030555	0.901	0.831
86688	52323	LNGH	SRX1254128	71669519	143339038	67272724	3117412	65666414	3016076	63762884	2872498	1679951	0.927	0.866
86689	52336	LNGH	SRX1254133	49810006	99620012	45382499	1896140	44225863	1826118	42132693	1699927	976238	0.900	0.826
86690	52349	LNGH	SRX1254138	46308545	92617090	42110736	1731249	40845147	1670292	38976619	1555570	1042514	0.896	0.819
86691	52362	LNGH	SRX1254143	50067717	100135434	45518671	1837494	44290433	1774343	42335465	1653799	999304	0.897	0.826
86692	52375	LNGH	SRX1254148	39486762	78973524	35667327	1545918	34587254	1483382	32917767	1377558	783364	0.890	0.814
86693	52386	LNGH	SRX1254153	43568305	87136610	39407037	1730793	38246424	1661858	36489150	1548802	964228	0.891	0.815
86694	52397	LNGH	SRX1254158	63539281	127078562	58001420	2561087	57001437	2507962	54326971	2306688	1202519	0.905	0.836
86695	52408	LNGH	SRX1254163	42779569	85559138	39114626	1716946	37965949	1656633	36385440	1553960	1006762	0.901	0.827
86696	52419	LNGH	SRX1254168	39325495	78650990	35848324	1465765	34765069	1412196	33214586	1328054	874510	0.898	0.822
86697	52430	LNGH	SRX1254173	54099742	108199484	49616972	2055282	48577176	2009634	46480823	1877140	1173978	0.908	0.837
86698	52441	LNGH	SRX1254178	66290811	132581622	61088490	2405522	59335418	2329524	56963402	2188061	1546980	0.908	0.836
86699	52452	LNGH	SRX1254183	43010668	86021336	39325858	1599238	38269028	1553757	36606013	1450205	1029222	0.902	0.827
86700	52463	LNGH	SRX1254189	65054122	130108244	59982883	2527424	58368560	2441307	55982958	2287170	1260530	0.910	0.841
86701	52474	LNGH	SRX1254194	52855065	105710130	49320388	2167818	48060785	2102813	46461710	1978792	1134400	0.921	0.858
86702	52485	LNGH	SRX1254199	48322715	96645430	44237870	1829808	43235179	1785034	41270514	1665818	1063963	0.905	0.832
86707	52540	LNGH	SRX1254204	43911954	87823908	40211251	1823149	39158473	1768975	37471770	1643391	965933	0.904	0.931
86708	52551	LNGH	SRX1254209	45687217	91374434	42095038	1709511	40977736	1664256	39332704	1552117	1046675	0.909	0.838
86709	52562	LNGH	SRX1254214	48998952	97997904	44964059	1979146	43441768	1902476	41620566	1767881	1151560	0.902	0.826
86710	52573	LNGH	SRX1254219	41748724	83497448	38007342	1717541	36922777	1657611	35221254	1525954	755015	0.897	0.826
86684	52241	LNGL	SRX1254109	50725614	101451228	46134469	1881965	44583000	1802192	42642429	1680948	900933	0.894	0.823
86685	52262	LNGL	SRX1254114	52089134	104178268	47105372	1927019	45238823	1838502	43275400	1714938	1130054	0.886	0.809
86686	52283	LNGL	SRX1254119	44690438	89380876	40265840	1649675	39135188	1593587	37201165	1479365	984992	0.888	0.810
86687	52304	LNGL	SRX1254124	48365543	96731086	43524885	1690850	42008037	1621563	40066233	1513780	978370	0.884	0.808
86688	52324	LNGL	SRX1254129	41269896	82539792	36724449	1636014	35388274	1553785	33595979	1424650	898633	0.874	0.792
86689	52337	LNGL	SRX1254134	47477760	94955520	42710439	1938058	41281150	1850265	39384273	1718543	1142141	0.885	0.805
86690	52350	LNGL	SRX1254139	56770579	113541158	51173415	2271744	49118104	2152510	46795954	1996338	1056871	0.883	0.806
86691	52363	LNGL	SRX1254144	48675315	97350630	43602857	1963121	41880784	1865820	39819069	1721876	1187629	0.878	0.794
86692	52376	LNGL	SRX1254149	44347111	88694222	39723088	1853704	38235163	1757382	36369239	1620147	956717	0.879	0.799
86693	52387	LNGL	SRX1254154	39198403	78396806	34945538	1492261	33223616	1395251	31549743	1290311	852284	0.870	0.783
86694	52398	LNGL	SRX1254159	55007277	110014554	49606288	2090801	48088642	2024556	45737733	1856553	1459036	0.888	0.805
86695	52409	LNGL	SRX1254164	46363325	92726650	41710435	1758722	39955558	1667798	38121108	1550826	990220	0.881	0.801
86696	52420	LNGL	SRX1254169	68483731	136967462	60834892	3337928	58363469	3185723	55221256	2880110	1451696	0.870	0.785
86697	52431	LNGL	SRX1254174	43839943	87679886	39657566	1568007	38426689	1516618	36628628	1404015	906628	0.891	0.815
86698	52442	LNGL	SRX1254179	48793932	97587864	43634160	1646341	41678120	1562127	39722903	1456237	979161	0.874	0.794
86699	52453	LNGL	SRX1254184	35769731	71539462	33048943	4441919	33072159	4419699	31576852	4263851	1296808	0.924	0.847
86700	52464	LNGL	SRX1254190	43367701	86735402	38941910	1668435	37273580	1584825	35553420	1473016	798569	0.879	0.801
86701	52475	LNGL	SRX1254195	42176824	84353648	38449660	1598656	36986477	1526440	35323730	1417894	844575	0.894	0.817
86702	52486	LNGL	SRX1254200	48060322	96120644	43661477	2027659	42559127	1967348	40440788	1812268	996779	0.897	0.821
86707	52541	LNGL	SRX1254205	51946585	103893170	46874009	2012601	45056845	1923295	42996555	1779090	1104761	0.885	0.806
86708	52552	LNGL	SRX1254210	47767192	95534384	43072812	1780151	41280237	1694510	39306400	1570032	971025	0.883	0.803
86709	52563	LNGL	SRX1254215	54066324	108132648	49618686	2027894	48059217	1952329	45985519	1822028	1105758	0.903	0.830
86710	52574	LNGL	SRX1254220	59666208	119332416	53753579	2444376	51787979	2380521	49158266	2140586	1276976	0.884	0.802
86684	52245	NLN	SRX1254111	49593719	99187438	44852336	2279796	42742598	2174658	40850550	1996106	1484244	0.883	0.794
86685	52266	NLN	SRX1254116	49524199	99048398	45022159	2108750	42425358	1979652	40510012	1835050	1927511	0.883	0.779
86686	52287	NLN	SRX1254121	42649244	85298488	38704815	1858884	37398644	1797360	35663828	1658742	1143964	0.892	0.809
86687	52308	NLN	SRX1254126	43412280	86824560	39217746	1849122	37343329	1758781	35681768	1622870	1283437	0.882	0.792

86688	52326	NLN	SRX1254131	37825490	75650980	33957199	1732324	32434765	1653876	30820186	1492183	1029863	0.878	0.788
86689	52339	NLN	SRX1254136	38691667	77383334	34442548	2012583	32907588	1905052	31114418	1738327	856964	0.870	0.782
86690	52352	NLN	SRX1254141	58837801	117675602	53174178	2642789	50275494	2485403	47928724	2271705	1732864	0.879	0.785
86691	52365	NLN	SRX1254146	52231288	104462576	46949235	2415166	44909482	2298326	42812922	2104857	1555331	0.879	0.790
86692	52378	NLN	SRX1254151	43111546	86223092	37863039	2230198	35525648	2133648	33397543	1847294	1424554	0.851	0.742
86693	52389	NLN	SRX1254156	39245719	78491438	35054946	1841118	32760210	1730315	31050499	1556382	1427454	0.864	0.755
86694	52400	NLN	SRX1254161	45885722	91771444	41490828	2090849	39968408	2025176	38017616	1846369	1925802	0.888	0.787
86695	52411	NLN	SRX1254166	42184969	84369938	37562091	2096278	35412506	1968994	33529747	1758674	1373961	0.865	0.762
86696	52422	NLN	SRX1254171	39399329	78798658	35524035	1941027	33735400	1846581	32106068	1686820	1410539	0.879	0.779
86697	52433	NLN	SRX1254176	43497222	86994444	39374866	1991835	37894508	1903968	36222762	1770098	1102941	0.888	0.807
86698	52444	NLN	SRX1254181	49505348	99010696	44254145	2264684	41801205	2137209	39768455	1950046	1360334	0.869	0.776
86699	52455	NLN	SRX1254187	47889637	95779274	42684393	2196479	40687644	2096418	38685112	1903384	1613895	0.870	0.774
86700	52466	NLN	SRX1254192	45418258	90836516	40930666	2027019	38777294	1912657	37032555	1768345	1423057	0.877	0.784
86701	52477	NLN	SRX1254197	50627528	101255056	45649334	2331730	43435589	2218329	41422109	2023107	1522455	0.880	0.788
86702	52488	NLN	SRX1254202	46038374	92076748	41302333	2056098	39456938	1975911	37440410	1796251	1552162	0.877	0.780
86707	52543	NLN	SRX1254207	62004953	124009906	55570924	2853358	52725110	2721831	50110792	2465697	1932740	0.873	0.777
86708	52554	NLN	SRX1254212	56584644	113169288	50600273	2503236	48437670	2428088	46000807	2160824	1518071	0.875	0.786
86709	52565	NLN	SRX1254217	75691178	151382356	68772997	3534075	66240831	3437067	63219211	3093243	1944640	0.892	0.810
86710	52576	NLN	SRX1254222	65386456	130772912	58596924	3335969	56290386	3290496	53438430	2918077	1506554	0.879	0.794
86684	52255	PGT	SRX1254112	49512059	99024118	46170916	2306174	45240806	2264042	43436723	2113492	1500881	0.923	0.847
86685	52276	PGT	SRX1254117	46934345	93868690	43934080	2103714	43228549	2066818	41609929	1946464	1375763	0.929	0.857
86686	52297	PGT	SRX1254122	49767800	99535600	47062717	2411516	46571983	2387857	45074200	2265962	1285114	0.941	0.880
86687	52318	PGT	SRX1254127	36439761	72879522	33788967	1727872	33203419	1696015	31911591	1584382	957913	0.919	0.849
86688	52331	PGT	SRX1254132	43513248	87026496	40712018	2172202	39690536	2116076	38155439	1962030	1271270	0.924	0.848
86689	52344	PGT	SRX1254137	40548522	81097044	36402279	1924326	35847810	1886990	33987911	1728690	1101112	0.891	0.811
86690	52357	PGT	SRX1254142	87163487	174326974	78679045	3837155	76800823	3730109	73115202	3456259	2773027	0.892	0.807
86691	52370	PGT	SRX1254147	50845820	101691640	45677743	2210616	44678201	2171061	42363980	1995664	1224923	0.889	0.809
86692	52383	PGT	SRX1254152	57791171	115582342	52397399	2715802	51407392	2663992	48884033	2447464	1630519	0.898	0.818
86693	52394	PGT	SRX1254157	68958662	137917324	62819524	3191765	61499730	3126181	58556013	2877956	2291506	0.901	0.816
86694	52405	PGT	SRX1254162	53858490	107716980	48589945	2359116	46433560	2247422	43829645	2058764	1616040	0.882	0.784
86695	52416	PGT	SRX1254167	65331932	130663864	59457548	2956645	58543248	2900705	55915356	2696007	2043358	0.903	0.825
86696	52427	PGT	SRX1254172	44579587	89159174	40521282	1875576	39869291	1844836	38053335	1713875	1224204	0.902	0.826
86697	52438	PGT	SRX1254177	38686392	77372784	35769184	1749870	35207420	1732870	33720717	1600232	1098043	0.917	0.843
86698	52449	PGT	SRX1254182	80862505	161725010	76462146	3622386	72975539	3484246	70587496	3244966	2415915	0.924	0.843
86699	52460	PGT	SRX1254188	47336445	94672890	44684812	2056428	43655056	2016615	42223914	1897759	1576363	0.933	0.859
86700	52471	PGT	SRX1254193	36854928	73709856	34758319	1589103	33759711	1544616	32631459	1463734	1144593	0.930	0.854
86701	52482	PGT	SRX1254198	41262229	82524458	39073859	1900563	38264776	1869029	37018633	1748600	1208603	0.937	0.868
86702	52493	PGT	SRX1254203	50962427	101924854	47756074	2281498	47140620	2268895	45424058	2115301	1503644	0.931	0.862
86707	52548	PGT	SRX1254208	44666039	89332078	41003970	2142971	40566002	2146457	38747876	1956002	1248189	0.913	0.840
86708	52559	PGT	SRX1254213	36718231	73436462	33635980	1769243	33196133	1766408	31642148	1602918	1032893	0.910	0.834
86709	52570	PGT	SRX1254218	61676571	123353142	56551877	2901007	56014564	2904895	53456162	2656540	1672444	0.913	0.840
86710	52581	PGT	SRX1254223	89276498	178552996	81550578	4168495	80222747	4136370	76605333	3783824	2378091	0.906	0.831
86684	52243	RLN	SRX1254110	49846558	99693116	45923239	2261646	44221027	2166426	42489119	2032607	1643815	0.904	0.819
86685	52264	RLN	SRX1254115	48104151	96208302	44075579	2125261	42332856	2024039	40638273	1894204	1550796	0.898	0.813
86686	52285	RLN	SRX1254120	57098483	114196966	52394459	2544713	51081557	2474562	48916316	2308562	2135942	0.906	0.819
86687	52306	RLN	SRX1254125	39749184	79498368	36413965	1846631	34781986	1749839	33333010	1636482	1357809	0.896	0.804
86688	52325	RLN	SRX1254130	39987352	79974704	36205207	1944282	34840255	1871110	33187551	1697834	1214183	0.888	0.800
86689	52338	RLN	SRX1254135	44802080	89604160	40700578	2251943	39223105	2152716	37441944	1984861	1336487	0.892	0.806
86690	52351	RLN	SRX1254140	39170879	78341758	35658613	1903744	34284856	1812528	32765998	1678093	1103463	0.893	0.808
86691	52364	RLN	SRX1254145	45324948	90649896	41284906	2111122	39649554	2021411	37874416	1866006	1393407	0.893	0.805
86692	52377	RLN	SRX1254150	44911935	89823870	38918038	3154700	37295270	3090968	34721940	2612888	1406687	0.848	0.742
86693	52388	RLN	SRX1254155	45473651	90947302	40839281	2321413	38866607	2237883	36908789	1989670	1388438	0.876	0.781
86694	52399	RLN	SRX1254160	54728120	109456240	49719240	2449413	48172031	2368904	45966495	2172365	1826187	0.894	0.807

86695	52410	RLN	SRX1254165	51801494	103602988	45801081	2952517	43828906	2878762	41314473	2495646	1595316	0.865	0.767
86696	52421	RLN	SRX1254170	38812896	77625792	35467197	1809219	33615162	1703695	32142765	1586055	1599643	0.890	0.787
86697	52432	RLN	SRX1254175	49495171	98990342	45541665	2177014	44020653	2092271	42252904	1962626	1414543	0.905	0.825
86698	52443	RLN	SRX1254180	46150213	92300426	42452256	2057602	40706810	1958350	39053919	1838194	1418763	0.901	0.815
86699	52454	RLN	SRX1254186	49851654	99703308	45792811	2317781	44338185	2234488	42518694	2084720	1644649	0.904	0.820
86700	52465	RLN	SRX1254191	37908915	75817830	35028056	1883307	33625741	1800499	32336197	1698030	1766974	0.906	0.806
86701	52476	RLN	SRX1254196	49855437	99710874	45521461	2354906	43647667	2247872	41839935	2087917	1455591	0.894	0.810
86702	52487	RLN	SRX1254201	45035907	90071814	40917072	2292437	39545704	2231448	37663859	2042270	1590806	0.893	0.801
86707	52542	RLN	SRX1254206	47346053	94692106	43234780	2271748	41373022	2178936	39562588	2001561	1613049	0.894	0.802
86708	52553	RLN	SRX1254211	45692822	91385644	41723098	2130827	39761123	2016969	37980906	1867911	1440987	0.892	0.800
86709	52564	RLN	SRX1254216	55060577	110121154	50304104	2489700	48185805	2369064	46213632	2197071	1405678	0.894	0.814
86710	52575	RLN	SRX1254221	65324609	130649218	59940448	3015572	58330794	2930774	55763079	2694748	1923754	0.905	0.824

Lab_id: Sample Identification; Tissue_ID: Tissue Identification; LNGL: lung lesion; LNGH: healthy lung; RLN: retropharyngeal lymph node, NLN: nasopharyngeal lymph node; PGT: pharyngeal tonsil;
N. Reads: Number of reads; Left_mapped: number of left reads mapped; Left_MA: number of left read multi-aligned; Right_mapped: number of right reads mapped; Righ_MA: number of right read multi-aligned;
MA: number of multi-aligned reads; discordant_pair: number of reads mapped discordantly, overall_mapp: overall mapping; concordant_pair: number of pair of reads mapped concordantly.

Table S2. List of immune function related genes differentially expressed in different tissue combinations in response to challenge by BRDC pathogens.

Immune rela	Gene Name	Predicted Function	BRDC Pathogen	Tissues*
BPIFA1	BPI fold-containing family A member 1	lipid binding	BRSV	BLN-LNGL-PGT
C1QA	Complement C1q subcomponent subunit A	protein binding	BRSV	NLN-PGT-RLN
C1QB	Complement C1q subcomponent subunit B	protein homodimerization activity	BRSV	BLN-NLN-PGT-RLN
C1QBP	Complement component 1 Q subcomponent-binding pro	mitochondrial ribosome binding	BRSV	BLN-LNGL
C4BPA	C4b-binding protein alpha chain	poly(A) RNA binding	BRSV	LNGL
C9	Complement component C9	protein binding	BRSV	RLN
CD14	Monocyte differentiation antigen CD14 precursor	ipoteichoic acid binding	BRSV	BLN-LNGL-NLN
CFB	Complement factor B Complement factor B Ba fragment	hydrolase activity	BRSV	BLN-LNGL-NLN-RLN
CFD	Complement factor D	hydrolase activity	BRSV	LNGL-NLN-RLN
CHID1	Chitinase domain-containing protein 1	hydrolase activity	BRSV	BLN-NLN-RLN
CLEC5A	C-type lectin domain family 5 member A	carbohydrate binding	BRSV	BLN-LNGL-NLN-PGT
CLEC6A	C-type lectin domain family 6 member A	carbohydrate binding	BRSV	BLN-NLN-PGT-RLN
CRCP	DNA-directed RNA polymerase III subunit RPC9	DNA-directed RNA polymerase activ	BRSV	PGT-RLN
CYBA	Cytochrome b-245 light chain	protein heterodimerization activity	BRSV	BLN-NLN-RLN
DDX3X	ATP-dependent RNA helicase DDX3X	mRNA 5'-UTR binding	BRSV	BLN-PGT-RLN
DDX58	Uncharacterized protein	hydrolase activity	BRSV	BLN-LNGL-NLN-PGT-RLN
ECSIT	Evolutionarily conserved signaling intermediate in Toll pa	oxidoreductase activity	BRSV	BLN
FADD	Protein FADD	identical protein binding	BRSV	BLN-LNGL-NLN-RLN
FCN2	Ficolin-2	carbohydrate derivative binding	BRSV	BLN-NLN-RLN
IFIH1	Uncharacterized protein	hydrolase activity	BRSV	BLN-LNGL-NLN-PGT-RLN
IGJ	Immunoglobulin J chain precursor	peptidoglycan binding	BRSV	BLN-RLN
IPO7	Importin-7	histone binding	BRSV	NLN-PGT-RLN
IRAK1	Interleukin-1 receptor-associated kinase 1	transferase activity	BRSV	LNGL
IRF1	Interferon regulatory factor 1	sequence-specific DNA binding	BRSV	LNGL-NLN
IRF3	Interferon regulatory factor 3	transcription regulatory region DNA	BRSV	BLN-LNGL-NLN-PGT-RLN
LGR4	Leucine-rich repeat-containing G-protein coupled recept	protein binding	BRSV	BLN-LNGL-RLN
MB21D1	Uncharacterized protein	cyclic-GMP-AMP synthase activity	BRSV	BLN-LNGL-PGT-RLN
MIF	Macrophage migration inhibitory factor	phenylpyruvate tautomerase activity	BRSV	BLN-LNGL
MX1	Interferon-induced GTP-binding protein Mx1 Interferon-i	GTP binding	BRSV	BLN-LNGL-NLN-PGT-RLN
MYD88	Myeloid differentiation primary response protein MyD88	TIR domain binding	BRSV	LNGL
NLRC5	Uncharacterized protein	nucleoside-triphosphatase activity	BRSV	LNGL
PGLYRP1	Peptidoglycan recognition protein 1	peptidoglycan binding	BRSV	BLN
PML	Uncharacterized protein	cobalt ion binding	BRSV	BLN-LNGL-NLN-PGT-RLN
POLR3A	DNA-directed RNA polymerase III subunit RPC1	metal ion binding	BRSV	BLN-LNGL
RSAD2	Radical S-adenosyl methionine domain-containing protei	4 iron	BRSV	BLN-LNGL-NLN-PGT-RLN
S100A12	Protein S100-A12	RAGE receptor binding	BRSV	BLN-RLN
S100A8	Protein S100-A8	RAGE receptor binding	BRSV	BLN-NLN-PGT-RLN
S100A9	Protein S100-A9	RAGE receptor binding	BRSV	BLN-NLN-PGT-RLN
SAMHD1	SAM domain and HD domain-containing protein 1	metal ion binding	BRSV	PGT-RLN
SFTPD	Pulmonary surfactant-associated protein D	carbohydrate binding	BRSV	NLN
SYK	Tyrosine-protein kinase SYK	transferase activity	BRSV	RLN
TLR2	Toll-like receptor 2 precursor	lipoteichoic acid binding	BRSV	BLN
TLR4	Toll-like receptor 4 precursor	protein binding	BRSV	BLN-LNGL-RLN
TMEM173	Transmembrane protein 173	cyclic-GMP-AMP binding	BRSV	LNGL
TRIM14	Uncharacterized protein	zinc ion binding	BRSV	RLN
TRIM15	Tripartite motif-containing protein 15	zinc ion binding	BRSV	LNGL
TRIM21	E3 ubiquitin-protein ligase TRIM21	metal ion binding	BRSV	BLN-LNGL-NLN-PGT-RLN
TRIM25	E3 ubiquitin/ISG15 ligase TRIM25	metal ion binding	BRSV	BLN-LNGL-NLN-PGT-RLN
TRIM26	Uncharacterized protein	metal ion binding	BRSV	BLN-LNGL-PGT-RLN
TRIM31	Uncharacterized protein	metal ion binding	BRSV	BLN
TRIM35	Uncharacterized protein	metal ion binding	BRSV	LNGL
TRIM56	E3 ubiquitin-protein ligase TRIM56	metal ion binding	BRSV	LNGL-PGT-RLN
TRIM62	Tripartite motif-containing protein 62	metal ion binding	BRSV	RLN
VNN1	Pantetheinase	hydrolase activity	BRSV	LNGL
AKIRIN2	Akirin-2	enzyme binding	BVDV	LNGL
APCS	Serum amyloid P-component	metal ion binding	BVDV	BLN
BCL10	B-cell lymphoma/leukemia 10	NF-kappaB binding	BVDV	LNGL-PGT
BPIFA1	BPI fold-containing family A member 1	lipid binding	BVDV	LNGL-PGT
C1QA	Complement C1q subcomponent subunit A	protein binding	BVDV	BLN-NLN-PGT-RLN
C1QB	Complement C1q subcomponent subunit B	protein homodimerization activity	BVDV	BLN-NLN-PGT-RLN
C1QBP	Complement component 1 Q subcomponent-binding pro	mitochondrial ribosome binding	BVDV	PGT
C4BPA	C4b-binding protein alpha chain	poly(A) RNA binding	BVDV	LNGL-NLN-PGT
C9	Complement component C9	protein binding	BVDV	BLN-PGT
CD14	Monocyte differentiation antigen CD14 precursor	lipoteichoic acid binding	BVDV	BLN-NLN-PGT
CFB	Complement factor B Complement factor B Ba fragment	hydrolase activity	BVDV	BLN-LNGL-NLN-PGT-RLN

CFD	Complement factor D	hydrolase activity	BVDV	BLN-LNGL-RLN
CHGA	Chromogranin-A Vasostatin-1 Chromostatin Chromacin P	protein binding	BVDV	BLN
CHID1	Chitinase domain-containing protein 1	hydrolase activity	BVDV	BLN
CLEC6A	C-type lectin domain family 6 member A	carbohydrate binding	BVDV	LNGL
CRCP	DNA-directed RNA polymerase III subunit RPC9	DNA-directed RNA polymerase activ	BVDV	BLN-NLN-PGT-RLN
CYBA	Cytochrome b-245 light chain	protein heterodimerization activity	BVDV	BLN
DDX3X	ATP-dependent RNA helicase DDX3X	mRNA 5'-UTR binding	BVDV	BLN
DDX58	Uncharacterized protein	hydrolase activity	BVDV	NLN-PGT
FADD	Protein FADD	identical protein binding	BVDV	BLN
FCN2	Ficolin-2	carbohydrate derivative binding	BVDV	BLN-NLN-PGT-RLN
FGR	tyrosine-protein kinase Fgr	transferase activity	BVDV	BLN-NLN-PGT-RLN
GATA3	Trans-acting T-cell-specific transcription factor GATA-3	HMG box domain binding	BVDV	NLN
IFIH1	Uncharacterized protein	hydrolase activity	BVDV	LNGL-NLN
IGJ	Immunoglobulin J chain precursor	peptidoglycan binding	BVDV	BLN-PGT-RLN
IPO7	Importin-7	histone binding	BVDV	BLN
IRF1	Interferon regulatory factor 1	sequence-specific DNA binding	BVDV	NLN-RLN
LGR4	Leucine-rich repeat-containing G-protein coupled recepto	protein binding	BVDV	NLN-PGT
MALT1	Uncharacterized protein	protein self-association	BVDV	BLN
MB21D1	Uncharacterized protein	cyclic-GMP-AMP synthase activity	BVDV	BLN-NLN
MIF	Macrophage migration inhibitory factor	phenylpyruvate tautomerase activity	BVDV	NLN-PGT
MR1	Major histocompatibility complex class I-related gene pro	peptide antigen binding	BVDV	NLN-PGT
MX1	Interferon-induced GTP-binding protein Mx1 Interferon-i	GTP binding	BVDV	BLN-NLN-PGT
NLRC5	Uncharacterized protein	nucleoside-triphosphatase activity	BVDV	NLN-PGT
NOD2	Nucleotide-binding oligomerization domain-containing p	CARD domain binding	BVDV	NLN-PGT
PGLYRP1	Peptidoglycan recognition protein 1	peptidoglycan binding	BVDV	BLN-NLN-PGT
PML	Uncharacterized protein	cobalt ion binding	BVDV	PGT
POLR3A	DNA-directed RNA polymerase III subunit RPC1	metal ion binding	BVDV	BLN-LNGL-NLN-PGT-RLN
RSAD2	Radical S-adenosyl methionine domain-containing protei	4 iron	BVDV	BLN-NLN-PGT
S100A12	Protein S100-A12	RAGE receptor binding	BVDV	BLN-NLN-PGT-RLN
S100A8	Protein S100-A8	RAGE receptor binding	BVDV	BLN-NLN-PGT-RLN
S100A9	Protein S100-A9	RAGE receptor binding	BVDV	BLN-NLN-PGT-RLN
SAMHD1	SAM domain and HD domain-containing protein 1	metal ion binding	BVDV	NLN
SFTPD	Pulmonary surfactant-associated protein D	carbohydrate binding	BVDV	LNGL-NLN-PGT
SYK	Tyrosine-protein kinase SYK	transferase activity	BVDV	NLN-PGT
TLR10	Toll-like receptor 10	protein binding	BVDV	NLN
TLR2	Toll-like receptor 2 precursor	lipoteichoic acid binding	BVDV	PGT
TLR3	Toll-like receptor 3 precursor	protein binding	BVDV	BLN
TLR4	Toll-like receptor 4 precursor	protein binding	BVDV	BLN-NLN-PGT-RLN
TLR5	Toll-like receptor 5 precursor	protein binding	BVDV	NLN
TMEM173	Transmembrane protein 173	cyclic-GMP-AMP binding	BVDV	NLN
TOLLIP	Toll-interacting protein	Toll-like receptor binding	BVDV	BLN
TRIM25	E3 ubiquitin/ISG15 ligase TRIM25	metal ion binding	BVDV	PGT
TRIM28	Uncharacterized protein	chromo shadow domain binding	BVDV	NLN-PGT
TRIM62	Tripartite motif-containing protein 62	metal ion binding	BVDV	PGT
TRIM8	Probable E3 ubiquitin-protein ligase TRIM8	metal ion binding	BVDV	NLN
VNN1	Pantetheinase	hydrolase activity	BVDV	BLN-LNGL-NLN-PGT-RLN
APCS	Serum amyloid P-component	metal ion binding	BoHV-1	LNGL
BPIFA1	BPI fold-containing family A member 1	lipid binding	BoHV-1	LNGL-PGT
C1QA	Complement C1q subcomponent subunit A	protein binding	BoHV-1	NLN-RLN
C1QB	Complement C1q subcomponent subunit B	protein homodimerization activity	BoHV-1	BLN-NLN-PGT-RLN
C1QBP	Complement component 1 Q subcomponent-binding pro	mitochondrial ribosome binding	BoHV-1	BLN-LNGL
C4BPA	C4b-binding protein alpha chain	poly(A) RNA binding	BoHV-1	LNGL-PGT
CD14	Monocyte differentiation antigen CD14 precursor	lipoteichoic acid binding	BoHV-1	BLN-LNGL-NLN-PGT-RLN
CFB	Complement factor B Complement factor B Ba fragment	hydrolase activity	BoHV-1	BLN-LNGL-NLN-PGT-RLN
CFD	Complement factor D	hydrolase activity	BoHV-1	LNGL-NLN-PGT-RLN
CHGA	Chromogranin-A Vasostatin-1 Chromostatin Chromacin P	protein binding	BoHV-1	BLN-RLN
CHID1	Chitinase domain-containing protein 1	hydrolase activity	BoHV-1	BLN-RLN
CLEC5A	C-type lectin domain family 5 member A	carbohydrate binding	BoHV-1	LNGL-RLN
CLEC6A	C-type lectin domain family 6 member A	carbohydrate binding	BoHV-1	BLN-NLN-PGT-RLN
CRCP	DNA-directed RNA polymerase III subunit RPC9	DNA-directed RNA polymerase activ	BoHV-1	BLN-NLN-RLN
CYBA	Cytochrome b-245 light chain	protein heterodimerization activity	BoHV-1	BLN
DDX3X	ATP-dependent RNA helicase DDX3X	mRNA 5'-UTR binding	BoHV-1	BLN
DDX58	Uncharacterized protein	hydrolase activity	BoHV-1	BLN-LNGL-NLN-PGT-RLN
ECSIT	Evolutionarily conserved signaling intermediate in Toll pa	oxidoreductase activity	BoHV-1	BLN
FADD	Protein FADD	identical protein binding	BoHV-1	BLN-LNGL-RLN
FCN2	Ficolin-2	carbohydrate derivative binding	BoHV-1	NLN-PGT-RLN
FGR	Tyrosine-protein kinase Fgr	transferase activity	BoHV-1	BLN-LNGL-NLN-PGT-RLN
GATA3	Trans-acting T-cell-specific transcription factor GATA-3	HMG box domain binding	BoHV-1	NLN
IFIH1	Uncharacterized protein	hydrolase activity	BoHV-1	BLN-LNGL-NLN-PGT-RLN

IFNB1	Interferon beta-1	type I interferon receptor binding	BoHV-1	NLN
IFNW1	Interferon omega-1	type I interferon receptor binding	BoHV-1	PGT
IGJ	Immunoglobulin J chain precursor	peptidoglycan binding	BoHV-1	BLN-RLN
IPO7	Importin-7	histone binding	BoHV-1	BLN-PGT
IRAK1	Interleukin-1 receptor-associated kinase 1	transferase activity	BoHV-1	BLN-LNGL-RLN
IRF1	Interferon regulatory factor 1	sequence-specific DNA binding	BoHV-1	NLN-PGT-RLN
IRF3	Interferon regulatory factor 3	transcription regulatory region DNA	BoHV-1	BLN-NLN-PGT-RLN
LGR4	Leucine-rich repeat-containing G-protein coupled recepto	protein binding	BoHV-1	BLN-LNGL-NLN-RLN
LOC513706	Uncharacterized protein	type I interferon receptor binding	BoHV-1	NLN
LOC618947	Uncharacterized protein	type I interferon receptor binding	BoHV-1	NLN-PGT
LOC783912	Uncharacterized protein	type I interferon receptor binding	BoHV-1	NLN
MALT1	Uncharacterized protein	protein self-association	BoHV-1	BLN
MB21D1	Uncharacterized protein	cyclic-GMP-AMP synthase activity	BoHV-1	LNGL-NLN-PGT-RLN
MIF	Macrophage migration inhibitory factor	phenylpyruvate tautomerase activity	BoHV-1	LNGL-NLN-RLN
MR1	Major histocompatibility complex class I-related gene pro	peptide antigen binding	BoHV-1	RLN
MX1	Interferon-induced GTP-binding protein Mx1 Interferon-i	GTP binding	BoHV-1	BLN-LNGL-NLN-PGT-RLN
NLRC5	Uncharacterized protein	nucleoside-triphosphatase activity	BoHV-1	NLN-PGT
NOD2	Nucleotide-binding oligomerization domain-containing p	CARD domain binding	BoHV-1	PGT
PGLYRP1	Peptidoglycan recognition protein 1	peptidoglycan binding	BoHV-1	BLN
PML	Uncharacterized protein	cobalt ion binding	BoHV-1	BLN-LNGL-NLN-PGT-RLN
POLR3A	DNA-directed RNA polymerase III subunit RPC1	metal ion binding	BoHV-1	BLN-NLN
RSAD2	Radical S-adenosyl methionine domain-containing protei	4 iron	BoHV-1	BLN-LNGL-NLN-PGT-RLN
S100A12	Protein S100-A12	RAGE receptor binding	BoHV-1	BLN-LNGL-PGT-RLN
S100A8	Protein S100-A8	RAGE receptor binding	BoHV-1	BLN-LNGL-NLN-PGT-RLN
S100A9	Protein S100-A9	RAGE receptor binding	BoHV-1	BLN-LNGL-NLN-PGT-RLN
SAMHD1	SAM domain and HD domain-containing protein 1	metal ion binding	BoHV-1	BLN
SFTPD	Pulmonary surfactant-associated protein D	carbohydrate binding	BoHV-1	LNGL-PGT
SYK	Tyrosine-protein kinase SYK	transferase activity	BoHV-1	BLN-NLN-RLN
TICAM2	TIR domain-containing adapter molecule 2	phospholipid binding	BoHV-1	NLN-RLN
TLR10	Toll-like receptor 10	protein binding	BoHV-1	NLN-RLN
TLR2	Toll-like receptor 2 precursor	lipoteichoic acid binding	BoHV-1	BLN-LNGL-NLN-PGT-RLN
TLR3	Toll-like receptor 3 precursor	protein binding	BoHV-1	BLN-NLN
TLR4	Toll-like receptor 4 precursor	protein binding	BoHV-1	BLN-LNGL-NLN-PGT-RLN
TLR5	Toll-like receptor 5 precursor	protein binding	BoHV-1	NLN-PGT
TMEM173	Transmembrane protein 173	cyclic-GMP-AMP binding	BoHV-1	BLN-LNGL
TRIM14	Uncharacterized protein	zinc ion binding	BoHV-1	NLN-PGT
TRIM21	E3 ubiquitin-protein ligase TRIM21	metal ion binding	BoHV-1	LNGL-NLN-PGT-RLN
TRIM25	E3 ubiquitin/ISG15 ligase TRIM25	metal ion binding	BoHV-1	BLN-LNGL-NLN-PGT-RLN
TRIM26	Uncharacterized protein	metal ion binding	BoHV-1	BLN-NLN-PGT
TRIM56	E3 ubiquitin-protein ligase TRIM56	metal ion binding	BoHV-1	NLN
VNN1	Pantetheinase	hydrolase activity	BoHV-1	LNGL-NLN
BPIFA1	BPI fold-containing family A member 1	lipid binding	MANNHE	LNGL-NLN-PGT
C1QA	Complement C1q subcomponent subunit A	protein binding	MANNHE	RLN
C1QB	Complement C1q subcomponent subunit B	protein homodimerization activity	MANNHE	RLN
C1QBP	Complement component 1 Q subcomponent-binding pro	mitochondrial ribosome binding	MANNHE	BLN
C4BPA	C4b-binding protein alpha chain	poly(A) RNA binding	MANNHE	LNGL
CD14	Monocyte differentiation antigen CD14 precursor	lipoteichoic acid binding	MANNHE	LNGL
CFB	Complement factor B Complement factor B Ba fragment	hydrolase activity	MANNHE	BLN-LNGL-PGT
CFD	Complement factor D	hydrolase activity	MANNHE	RLN
CHID1	Chitinase domain-containing protein 1	hydrolase activity	MANNHE	BLN-RLN
CLEC5A	C-type lectin domain family 5 member A	carbohydrate binding	MANNHE	BLN-LNGL
CLEC6A	C-type lectin domain family 6 member A	carbohydrate binding	MANNHE	LNGL
CRCP	DNA-directed RNA polymerase III subunit RPC9	DNA-directed RNA polymerase activ	MANNHE	BLN
DDX58	Uncharacterized protein	hydrolase activity	MANNHE	BLN-RLN
FCN2	Ficolin-2	carbohydrate derivative binding	MANNHE	BLN-LNGL-RLN
FGR	Tyrosine-protein kinase Fgr	transferase activity	MANNHE	LNGL
GATA3	Trans-acting T-cell-specific transcription factor GATA-3	HMG box domain binding	MANNHE	BLN-LNGL
IFIH1	Uncharacterized protein	hydrolase activity	MANNHE	BLN-LNGL
IGJ	Immunoglobulin J chain precursor	peptidoglycan binding	MANNHE	BLN-LNGL-RLN
IRAK1	Interleukin-1 receptor-associated kinase 1	transferase activity	MANNHE	BLN
IRF1	Interferon regulatory factor 1	sequence-specific DNA binding	MANNHE	BLN
LGR4	Leucine-rich repeat-containing G-protein coupled recepto	protein binding	MANNHE	RLN
MALT1	Uncharacterized protein	protein self-association	MANNHE	LNGL
MB21D1	Uncharacterized protein	cyclic-GMP-AMP synthase activity	MANNHE	BLN
MIF	Macrophage migration inhibitory factor	phenylpyruvate tautomerase activity	MANNHE	LNGL
MX1	Interferon-induced GTP-binding protein Mx1 Interferon-i	GTP binding	MANNHE	BLN-RLN
NLRC5	Uncharacterized protein	nucleoside-triphosphatase activity	MANNHE	BLN
PGLYRP1	Peptidoglycan recognition protein 1	peptidoglycan binding	MANNHE	BLN-LNGL
PML	Uncharacterized protein	cobalt ion binding	MANNHE	BLN

RSAD2	Radical S-adenosyl methionine domain-containing protei	4 iron	MANNHE	BLN-LNGL-RLN
S100A12	Protein S100-A12	RAGE receptor binding	MANNHE	BLN-RLN
S100A8	Protein S100-A8	RAGE receptor binding	MANNHE	BLN-LNGL-PGT-RLN
S100A9	Protein S100-A9	RAGE receptor binding	MANNHE	BLN-LNGL-NLN-RLN
TLR10	Toll-like receptor 10	protein binding	MANNHE	BLN-LNGL
TLR3	Toll-like receptor 3 precursor	protein binding	MANNHE	BLN
TMEM173	Transmembrane protein 173	cyclic-GMP-AMP binding	MANNHE	LNGL
TRIM21	E3 ubiquitin-protein ligase TRIM21	metal ion binding	MANNHE	BLN
VNN1	Pantetheinase	hydrolase activity	MANNHE	LNGL
BPIFA1	BPI fold-containing family A member 1	lipid binding	MYCO	BLN-LNGL-NLN
C4BPA	C4b-binding protein alpha chain	poly(A) RNA binding	MYCO	LNGL-PGT
C9	Complement component C9	protein binding	MYCO	PGT
CD14	Monocyte differentiation antigen CD14 precursor	ipoteichoic acid binding	MYCO	LNGL
CFB	Complement factor B Complement factor B Ba fragment	hydrolase activity	MYCO	LNGL
DDX58	Uncharacterized protein	hydrolase activity	MYCO	NLN-PGT
FCN2	Ficolin-2	carbohydrate derivative binding	MYCO	NLN
IFIH1	Uncharacterized protein	hydrolase activity	MYCO	NLN
IGJ	Immunoglobulin J chain precursor	peptidoglycan binding	MYCO	LNGL
MIF	Macrophage migration inhibitory factor	phenylpyruvate tautomerase activity	MYCO	LNGL
MX1	Interferon-induced GTP-binding protein Mx1 Interferon-i	GTP binding	MYCO	PGT-RLN
PGLYRP1	Peptidoglycan recognition protein 1	peptidoglycan binding	MYCO	BLN
RSAD2	Radical S-adenosyl methionine domain-containing protei	4 iron	MYCO	BLN-NLN-PGT
S100A12	Protein S100-A12	RAGE receptor binding	MYCO	RLN
S100A8	Protein S100-A8	RAGE receptor binding	MYCO	BLN-LNGL-PGT-RLN
S100A9	Protein S100-A9	RAGE receptor binding	MYCO	BLN-LNGL-NLN-PGT-RLN
SFTPD	Pulmonary surfactant-associated protein D	carbohydrate binding	MYCO	LNGL
TLR10	Toll-like receptor 10	protein binding	MYCO	LNGL
TMEM173	Transmembrane protein 173	cyclic-GMP-AMP binding	MYCO	LNGL
VNN1	Pantetheinase	hydrolase activity	MYCO	LNGL-NLN

*Footnote for abbreviations: bronchial lymph node (BLN), retropharyngeal lymph node (RLN), nasopharyngeal lymph node (NLN), pharyngeal tonsil (PGT) and lung (LNGL). For LNGL tissue, we compared expression changes in uninfected control vs. samples with lung lesion.

Table S3. Immune function related genes differentially expressed between lesion and healthy lung tissue in response to challenge by different BRDC pathogens.

Gene Symbol	Gene Name	Predicted Function
**Pathogen: BRSV**		
BPIFA1	BPI fold-containing family A member 1	lipid binding
C4BPA	C4b-binding protein alpha chain	poly(A) RNA binding
CD14	Monocyte differentiation antigen CD14 precursor	lipoteichoic acid binding
CFB	Complement factor B Complement factor B Ba fra	hydrolase activity
DDX58	Uncharacterized protein	hydrolase activity
IFIH1	Uncharacterized protein	hydrolase activity
IRF1	Interferon regulatory factor 1	sequence-specific DNA binding
MB21D1	Uncharacterized protein	cyclic-GMP-AMP synthase activity
PML	Uncharacterized protein	cobalt ion binding
RSAD2	Radical S-adenosyl methionine domain-containing	4 iron
TLR3	Toll-like receptor 3 precursor	protein binding
TMEM173	Transmembrane protein 173	cyclic-GMP-AMP binding
TRIM21	E3 ubiquitin-protein ligase TRIM21	metal ion binding
TRIM25	E3 ubiquitin/ISG15 ligase TRIM25	metal ion binding
VNN1	Pantetheinase	hydrolase activity
**Pathogen: BVDV**		
BPIFA1	BPI fold-containing family A member 1	lipid binding
DDX58	Uncharacterized protein	hydrolase activity
IFIH1	Uncharacterized protein	hydrolase activity
IRF1	Interferon regulatory factor 1	sequence-specific DNA binding
RSAD2	Radical S-adenosyl methionine domain-containing	4 iron
VNN1	Pantetheinase	hydrolase activity
CFD	Complement factor D	hydrolase activity
CLEC6A	C-type lectin domain family 6 member A	carbohydrate binding
FCN2	Ficolin-2	carbohydrate derivative binding
MX1	Interferon-induced GTP-binding protein Mx1 Inter	GTP binding
S100A12	Protein S100-A12	RAGE receptor binding
S100A8	Protein S100-A8	RAGE receptor binding
S100A9	Protein S100-A9	RAGE receptor binding
SFTPD	Pulmonary surfactant-associated protein D	carbohydrate binding
TLR2	Toll-like receptor 2 precursor	lipoteichoic acid binding
**Pathogen: BoHV-1**		
APCS	Serum amyloid P-component	metal ion binding
**Pathogen: MANNHE**		
VNN1	Pantetheinase	hydrolase activity
CLEC6A	C-type lectin domain family 6 member A	carbohydrate binding
FCN2	Ficolin-2	carbohydrate derivative binding
S100A8	Protein S100-A8	RAGE receptor binding
S100A9	Protein S100-A9	RAGE receptor binding
CD14	Monocyte differentiation antigen CD14 precursor	lipoteichoic acid binding
TMEM173	Transmembrane protein 173	cyclic-GMP-AMP binding
CLEC5A	C-type lectin domain family 5 member A	carbohydrate binding
GATA3	Trans-acting T-cell-specific transcription factor GA	HMG box domain binding
MIF	Macrophage migration inhibitory factor	phenylpyruvate tautomerase activity
NOD2	Nucleotide-binding oligomerization domain-conta	CARD domain binding
TLR10	Toll-like receptor 10	protein binding
TRIM14	Uncharacterized protein	zinc ion binding

Pathogen: MYCO

VNN1	Pantetheinase	hydrolase activity
FCN2	Ficolin-2	carbohydrate derivative binding
S100A8	Protein S100-A8	RAGE receptor binding
S100A9	Protein S100-A9	RAGE receptor binding
CD14	Monocyte differentiation antigen CD14 precursor	lipoteichoic acid binding
BPIFA1	BPI fold-containing family A member 1	lipid binding
C4BPA	C4b-binding protein alpha chain	poly(A) RNA binding
CFB	Complement factor B Complement factor B Ba fra	hydrolase activity
